# Assessing Cu_3_BiS_3_ for Thin-Film Photovoltaics: A Systematic DFT Study Comparing LCAO and PAW Across Multiple Functionals

**DOI:** 10.3390/ma18061213

**Published:** 2025-03-08

**Authors:** Carlos O. Amorim, Sivabalan M. Sivasankar, António F. da Cunha

**Affiliations:** Physics Department and i3N, University of Aveiro, Campus de Santiago, 3810-193 Aveiro, Portugal; sivabalanms@ua.pt (S.M.S.); antonio.cunha@ua.pt (A.F.d.C.)

**Keywords:** DFT, photovoltaics, ab-initio calculations, Cu_3_BiS_3_, solar cells

## Abstract

Cu_3_BiS_3_ (CBS) has emerged as a promising earth-abundant absorber for thin-film photovoltaics, offering a sustainable alternative to conventional technologies. However, ab initio studies on its optoelectronic properties remain scarce and often yield contradictory results. This study systematically examines the influence of two density functional theory (DFT) methodologies, linear combination of atomic orbitals (LCAO) and projector augmented wave (PAW), on the structural and electronic properties of CBS, aiming to establish a reliable computational framework for future research. With this in mind, we also assessed the impact of a wide range of exchange-correlation (XC) functionals within both methods, including 6 from the local density approximation (LDA) family (HL, PW, PZ, RPA, Wigner, XA), 10 from the generalized gradient approximation (GGA) family (BLYP, BP86, BPW91, GAM, KT2, PBE, PBEsol, PW91, RPBE, XLYP), 2 meta-GGA functionals (SCAN, R2SCAN), and the hybrid HSE06 functional. Both LCAO and PAW consistently predict an indirect bandgap for CBS across all XC functionals, aligning with most previous DFT studies but contradicting experimental reports of a direct transition. The LDA and meta-GGA functionals systematically underestimated the CBS bandgap (<1 eV), with further reductions upon structural relaxation. GGA functionals performed better, with BLYP and XLYP yielding the most experimentally consistent results. The hybrid HSE06 functional substantially overestimated the bandgap (1.9 eV), with minimal changes after relaxation. The calculated hole and electron effective masses reveal strong anisotropy along the X, Y, and Z crystallographic directions. Additionally, CBS exhibits an intrinsic p-type nature, as the Fermi level consistently lies closer to the valence band maximum across all methods and functionals. However, the PAW method generally predicted more accurate lattice parameters than LCAO; the best agreement with experimental values was achieved using the PW91 (1.2% deviation) and HSE06 (0.9% deviation) functionals within LCAO. Based on these findings, we recommend the PW91 functional with LCAO for structural optimizations in large supercell studies of CBS dopants and/or defects and BLYP/XLYP for electronic properties.

## 1. Introduction

As humanity is confronted with both energy insecurity and climate change, transitioning to renewable energy sources is not just imperative but urgent. Among renewable technologies, solar energy stands out as a scalable and sustainable solution to meet the ever-growing global energy demand while supporting decarbonization efforts and mitigating climate impacts [1,2,3,4]. The decentralization of solar energy deployment through photovoltaic (PV) panels, integrated into various environments, is key to enabling their widespread adoption [5,6,7,8,9]. Thin-film (TF) PV technologies, in particular, can be deposited in non-traditional substrates (e.g., flexible substrates), hence offering the versatility needed for these decentralized applications. This allows integration onto diverse surfaces, including building facades, windows with semi-transparent solar coatings, rooftops where traditional panels are impractical, and urban infrastructure such as noise barriers along highways [10,11,12,13,14,15,16]. Additionally, lightweight and flexible TF PVs enable deployment in wearable electronics, self-powered sensors for Internet of Things (IoT) applications, portable chargers for off-grid use, and vehicle-integrated photovoltaics in electric cars and buses. Such innovations expand solar energy accessibility, making it viable in locations where conventional rigid panels would be unsuitable [10,11,12,13,14,15,16].

Despite their potential, current TF PV technologies, such as CdTe and Cu(In,Ga)Se_2_ (CIGS), face significant challenges due to their reliance on critical or toxic elements, which limit scalability, affordability, and environmental sustainability [17,18]. Addressing these concerns requires developing alternative absorber materials that are abundant, non-toxic, and environmentally friendly, a set of requirements that Cu_3_BiS_3_ (CBS) appears well-suited to fulfill [19,20].

Cu_3_BiS_3_, a ternary chalcogenide with the orthorhombic Wittichenite phase, has garnered attention for its stability, low toxicity, and use of Earth-abundant elements [19,20,21]. These properties position CBS as a promising candidate for sustainable and affordable TF PV applications. Furthermore, CBS exhibits exceptional optical properties, including a tunable bandgap ranging from 1.0 to 1.8 eV [21,22,23,24,25,26,27,28,29,30,31,32]. This flexibility enables its optimization to the optimal value of 1.34 eV, as predicted by the Shockley–Queisser limit [33,34]. Additionally, its high absorption coefficient $\left(>10^{5}\;{cm}^{-1}\right)$ surpasses those of established materials like CIGS and CdTe, further enhancing its potential as an absorber layer in TF PVs [21,22,29,30,31,32].

However, CBS technology is still in its infancy, with the few experimental device-level studies to date reporting power conversion efficiencies (PCEs) not exceeding 1.7% [21,35], far below the performance of established TF PV technologies (with PCEs >20% [36,37,38,39]). This lack of maturity, primarily due to insufficient research and development investments, is likely rooted in the predominant focus on organic-inorganic perovskite solar cells, which have dominated recent photovoltaic research endeavors.

Despite the limited PCE demonstrated in the device-level research, CBS’s potential is evident. Simulations using the solar cell capacitance simulator SCAPS-1D [40,41,42] have shown that with proper band alignment, particularly through optimized n-type buffer layers, it is theoretically possible to achieve PCEs exceeding 20% [40], on par with CdTe and CIGS [36,37,38,39]. Moreover, unlike crystalline silicon, which dominates the market but lacks integration flexibility due to being >100× thicker, CBS-based TF PVs could seamlessly integrate into various architectural surfaces, enhancing their overall functionality [10,11,12,13,43].

To accelerate the development of CBS, density functional theory (DFT) is a critical computational tool. DFT not only facilitates the prediction of unexplored materials but also provides deep insights into the physical mechanisms underlying observed phenomena [44,45,46,47,48,49,50,51,52,53]. Notwithstanding, DFT calculations depend heavily on the adopted methods and chosen exchange-correlation functionals. Widely used DFT methods include linear combination of atomic orbitals (LCAO) [54], pseudopotential plane-wave (PP-PW) [55], projector-augmented wave (PAW) [56], and linearized augmented plane wave (LAPW) [57,58]. Each method comes with trade-offs in accuracy and computational cost. Similarly, exchange-correlation functionals range from the Local Density Approximation (LDA) and the semi-local Generalized Gradient Approximation (GGA) to the more advanced meta-GGA and hybrid functionals. While higher-level functionals like the hybrid HSE06 functional can provide improved accuracy, they are computationally expensive, and it is not always guaranteed that they are the most suitable for all systems.

For CBS, the limited DFT studies available have employed diverse approaches, including PP-PW, PAW, and LAPW methods, alongside different functionals. Additionally, some studies constrained the unit cell to experimental values, while others allowed full structure optimization. These differing methodologies, functionals, and unit cells have resulted in some inconsistencies across the reported findings [59,60,61,62,63,64,65,66]. Despite these challenges, these DFT investigations have been instrumental in predicting CBS’s high absorption coefficient and identifying band alignment challenges with the commonly used CdS buffer layer [21,23,61]. These theoretical predictions were later validated through experimental measurements and SCAPS simulations [21,23,40,41,61], which revealed that replacing the CdS buffer with a material offering better alignment could enhance PCEs from 7.6% to more than 20%. This underscores the pivotal role of computational approaches in advancing material optimization.

Advancing photovoltaic technologies based on novel absorber materials requires a deep understanding of their properties and the underlying physical mechanisms to enable precise control and optimization. In particular, achieving p-type conductivity is crucial, as it involves identifying the defects (such as vacancies) or dopants that effectively modify the semiconductor’s electronic structure. This control is essential not only to tune charge carrier density and mobility but also to enable bandgap engineering that ensures optimal alignment with the other layers of the solar cell. DFT offers a pathway to investigate doping, vacancies, and other band engineering strategies essential for improving CBS’s PCE. While high-level methods such as PAW with hybrid functionals like HSE06 provide detailed electronic and optical property descriptions (such as the one provided by Kumar et al. and Whittles et al. [60,61]), they are computationally prohibitive for the large supercells required to study defect chemistry and charge carrier dynamics. Even then, deviations from experimental results persist, emphasizing the need for a systematic exploration of the adopted computational approaches.

This work presents a comprehensive study comparing two DFT methods (LCAO and PAW) and various functional families, including LDA, GGA, meta-GGA, and hybrid, with multiple parameterizations. By systematically evaluating these approaches, we aim to understand their influence on CBS’s predicted properties and propose efficient, reliable computational strategies for future studies. Our findings provide a roadmap for selecting appropriate methods and functionals, balancing accuracy and computational cost, and accelerating the development of CBS-based TF PVs.

## 2. Computational Details

DFT [67,68] calculations were performed using the QuantumATK W-2024.09-SP1 software package [69]. Two distinct computational approaches were employed—the Linear Combination of Atomic Orbitals (LCAO) method [54] and the Projector-Augmented Wave (PAW) method [56]—to enable a comprehensive evaluation of methodological influences on the properties of Cu_3_BiS_3_.

For the LCAO method, a medium basis set was utilized with norm-conserving pseudopotentials from the PseudoDojo library [70] with a density mesh cut-off of 2900 eV. The PAW method employed PseudoDojo PAW potentials with a plane-wave cutoff energy of 500 eV.

A wide range of exchange-correlation (XC) functionals was explored to ensure a comprehensive analysis. The XC functionals were selected from all predefined options available in the QuantumATK package, as they were compiled to provide a representative set widely used across different system types and property-specific applications. For the LCAO method, functionals from the Local Density Approximation (LDA), Generalized Gradient Approximation (GGA), meta-GGA, and hybrid families were considered. The LDA functionals included six parameterizations: Hedin–Lundqvist (HL) [71], Perdew–Wang (PW) [72], Perdew–Zunger (PZ) [73], Random Phase Approximation (RPA) [74,75], Wigner [76,77], and Slater’s Xα (XA) [78]. The GGA functionals comprised 10 parameterizations: Becke–Lee–Yang–Parr (BLYP) [79,80,81], Becke–Perdew 86 (BP86) [79,82], Becke-Perdew–Wang 91 (BPW91) [79,83,84], Gradient Approximation for Molecules (GAM) [85], Keal–Tozer 2 (KT2) [86], Perdew–Burke–Ernzerhof (PBE) [87], Perdew–Burke–Ernzerhof for solids (PBEsol) [88], Perdew–Wang 91 (PW91) [83,84], Revised Perdew–Burke-Ernzerhof (RPBE) [89], and Xu–Lee–Yang–Parr (XLYP) [90]. Meta-GGA functionals included the Strongly Constrained and Appropriately Normed (SCAN) functional [91] and its revised version (R2SCAN) [92]. The Heyd–Scuseria–Ernzerhof (HSE06) hybrid functional [93,94] was also investigated. For the PAW method, only the LDA and GGA families were explored, using the same parameterizations as in the LCAO calculations.

Two types of structures were considered. Initially, fully converged self-consistent field (SCF) calculations were performed without structure optimization, using the orthorhombic Wittichenite (space group *P*2_1_2_1_2_1_) unit cell constrained to the experimental lattice parameters reported by Kocman et al. (a=7.723 Å, b=10.395 Å, c=6.716 Å, and α=β=γ=90°) [95]. The energy tolerance for convergence in these SCF calculations was set to $10^{-5}\;eV$ for LCAO and $10^{-6}\;eV$ for PAW.

In addition to constrained unit cell calculations, full structure optimizations were performed for each method and functional. These optimizations employed the Quasi-Newton Limited-Memory Broyden–Fletcher–Goldfarb–Shanno (LBFGS) method with Hellmann–Feynman forces, pressure, and energy tolerances of $0.01\;eV{Å}^{-1},\;0.1\;GPa,\;\mathrm{and}\;10⁵\;eV$, respectively.

The Brillouin zone was sampled using a Γ-centered Monkhorst-Pack k-mesh. For both structure optimization and SCF calculations, a k-point mesh density of 7 Å×7 Å×7 Å was employed, corresponding to at least 6×5×7 k-points. For electronic structure analysis, including density of states (DoS) and band structure calculations, a denser k-point mesh density of 12 Å×12 Å×12 Å was used, resulting in at least 11×9×12 k-points.

## 3. Results and Discussion

### 3.1. Experimental Wittichenite Structure

In this work, we investigate the influence of different DFT approaches on the optoelectronic properties of CBS, examining these effects across various levels of theoretical sophistication. At a broader level, we compare two widely used DFT methodologies: the LCAO and the PAW methods. Furthermore, we assess the impact of incrementally more advanced exchange-correlation (XC) functional families, beginning with LDA and progressing to GGA. For the LCAO method, we extend this analysis to include meta-GGA and hybrid functionals. Additionally, we evaluate which specific functionals and parameterizations within the LDA, GGA, and meta-GGA families are most suitable for studying the CBS system.

To ensure a consistent basis for comparison, all calculations were performed using the same unit cell, based on the orthorhombic Wittichenite structure of CBS (space group *P*2_1_2_1_2_1_), with lattice parameters and atomic positions determined experimentally by Kocman et al. [95]. Fixing the unit cell across SCF calculations eliminates structural variability, ensuring that differences in the predicted optoelectronic properties arise solely from variations in the DFT methodology or XC functionals rather than from differences in structural relaxation.

#### 3.1.1. LCAO Calculations with Experimental Structure

The LCAO method is a computational approach for solving the Kohn–Sham equations of DFT, where electronic wavefunctions are expressed as a linear combination of pre-defined atomic-like orbitals. This method offers computational efficiency, making it particularly suitable for systems with predominantly localized states and larger supercells. While LCAO is generally regarded as less accurate than higher-level methods, such as the PAW method, its lower computational cost enables the use of more sophisticated exchange-correlation functionals, such as meta-GGA and hybrid functionals. These advanced functionals can help mitigate some of the inaccuracies typically associated with LCAO, improving the reliability of the results.

**Figure 1:**
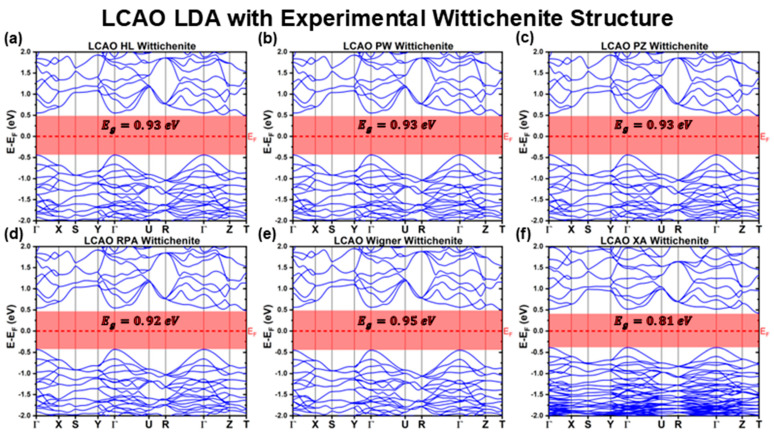
Cu_3_BiS_3_ band structures calculated using the LCAO method for different LDA functionals, considering the experimental Wittichenite structure.

**Figure 2:**
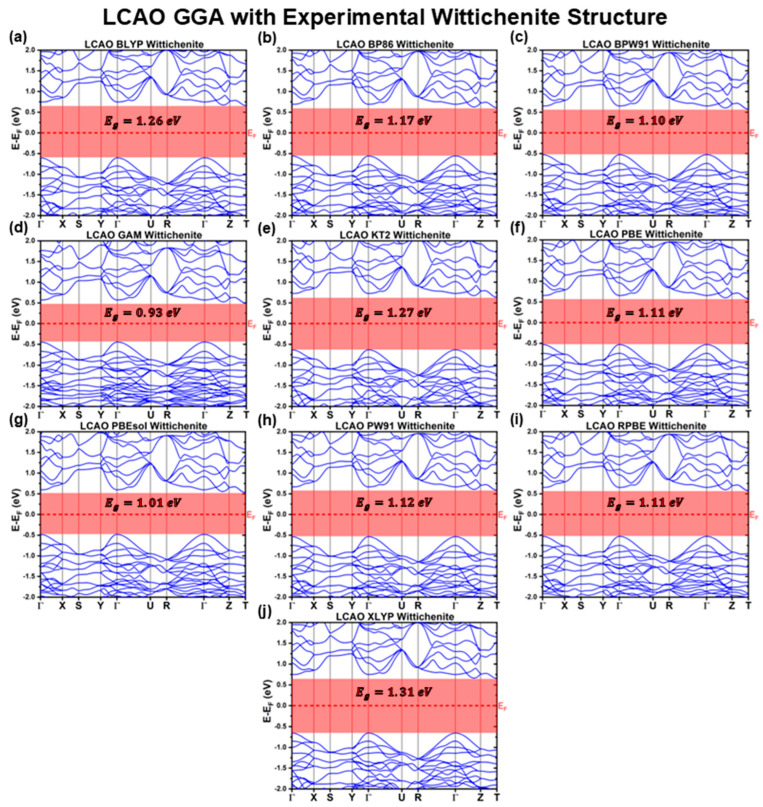
Cu_3_BiS_3_ band structures calculated using the LCAO method for different GGA functionals, considering the experimental Wittichenite structure.

**Figure 3:**
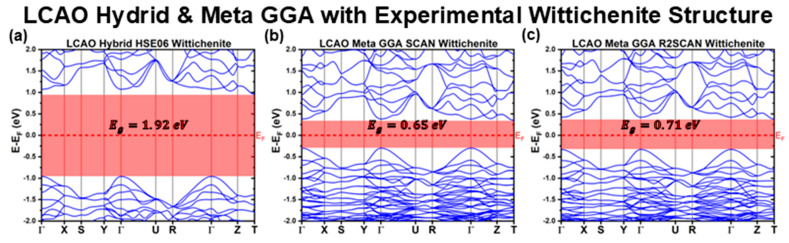
Cu_3_BiS_3_ band structures calculated using the LCAO method for different meta-GGA and Hybrid functionals, considering the experimental Wittichenite structure.

Figure 1, Figure 2 and Figure 3 illustrate the band structures calculated using the LCAO method for all the tested functionals. From these results, we extracted key optoelectronic properties such as the bandgap energy ($E_{g}$), hole and electron effective masses ($m_{h}^{*}$ and $m_{e}^{*}$, respectively), and the relative positions of the conduction band minimum (CBM) and valence band maximum (VBM) with respect to the Fermi level ($E_{F}$). These properties are comprehensively summarized in Table 1 and Table 2.

The band structures calculated using the LCAO method consistently predict that CBS has an indirect bandgap, with the VBM located at the Γ point of the Brillouin zone and the CBM at the T point. However, for all LCAO functionals, the conduction band exhibits a local energy minimum at the Γ point, with an energy closely approaching that of the CBM at T. This results in a direct $E_{g}$ that is close to the indirect $E_{g}$ (${\left[E_{g}\right]}_{direct}\approx {\left[E_{g}\right]}_{indirect}$).

For all LDA functionals, the predicted indirect $E_{g}$ values fall below the 1 eV threshold, which is at the lower end of the experimentally reported range of ${\left[E_{g}\right]}_{exp}\in \left[1;1.8\right]\;\mathrm{eV}$ for CBS [21,22,23,24,25,26,27,28,29,30,31,32]. Most LDA functionals yield $E_{g}\approx 0.93\;\mathrm{eV}$, except for the XA functional, which predicts an even lower $E_{g}$ of 0.81 eV. A closer inspection of the band structures shown in Figure 1 reveals that the band structures derived from the HL, PW, PZ, RPA, and Wigner XC functionals are nearly identical. In contrast, the XA functional exhibits a slightly different band structure, characterized by more compact and flatter bands, particularly below −1 eV.

To analyze the charge carrier dynamics, the effective masses of holes and electrons ($m_{h}^{*}$ and $m_{e}^{*}$, respectively) were estimated at the respective band edges (VBM and CBM) assuming the parabolic energy dispersion relationship described in Equation (1):

(1) $$E\left(k\right)=E_{0}+\frac{\hbar^{2}k^{2}}{2m^{*}},$$

where $E\left(k\right)$ is the energy of the charge carrier as a function of the wavevector k for a given band, $E_{0}$ is a constant that shifts the band to the respective band edge, ℏ is the reduced Planck constant, and $m^{*}$ is the effective mass of the charge carrier. From equation 1, $m^{*}$ can be derived as a function of the second derivative of $E\left(k\right)$, as shown in Equation (2):

(2) $$m^{*}=\frac{\hbar^{2}}{\left(\frac{\partial^{2}E\left(k\right)}{\partial k^{2}}\right)}.$$

Using this approach, the $m_{e}^{*}$ and $m_{h}^{*}$ were calculated at the CBM and VBM, respectively. For the hole charge carrier, its effective mass was calculated at the Γ point along the X, Y, and Z directions, designated as ${\left(m_{h}^{*}\right)}_{\Gamma_{X}}$, ${\left(m_{h}^{*}\right)}_{\Gamma_{Y}}$ and ${\left(m_{h}^{*}\right)}_{\Gamma_{Z}}$, respectively.

The effective mass results in Table 2 highlight the strong similarity among the HL, PW, PZ, RPA, and Wigner XC functionals, which yield nearly identical values. In contrast, the XA functional deviates significantly, particularly in predicting ${\left(m_{h}^{*}\right)}_{\Gamma_{Y}}$, which is nearly twice as large as the corresponding values from the other LDA functionals. A notable trend observed across all functionals is the anisotropy of hole effective masses at the Γ point. For HL, PW, PZ, RPA, and Wigner, ${\left(m_{h}^{*}\right)}_{\Gamma_{Y}}$ is consistently around half the free electron mass ($m_{0}$), while ${\left(m_{h}^{*}\right)}_{\Gamma_{X}}$ and ${\left(m_{h}^{*}\right)}_{\Gamma_{Z}}$ are approximately ${(-1.4\pm 0.1)m}_{0}$. The XA functional maintains this anisotropic behavior but predicts overall higher hole effective masses and introduces additional disparity between the X and Z directions.

From Figure 2, it is evident that the band structures calculated using most GGA functionals are qualitatively similar, displaying nearly identical profiles with minor differences in relative energy levels between bands. These subtle variations result in different predicted $E_{g}$. Interestingly, the band profiles of the GGA functionals are not only consistent among themselves but also closely resemble those obtained using LDA functionals, excluding the XA functional. This observation is supported by the calculated effective masses, which are ${\left(m_{h}^{*}\right)}_{\Gamma_{X}}={\left(m_{h}^{*}\right)}_{\Gamma_{Z}}=\left(-1.4\pm 0.1\right)m_{0};{\left(m_{h}^{*}\right)}_{\Gamma_{y}}=\left(-0.4\pm 0.1\right)m_{0}\;\mathrm{and}\;m_{e}^{*}=0.6m_{0}$ for all the GGA and LDA XC functionals, again excluding XA.

The LCAO calculations considering GGA XC functionals predict higher $E_{g}$ values that spread in a wider range compared to their LDA counterparts with ${\left[E_{g}\right]}_{\mathrm{GGA}}^{{\mathrm{LCAO}}_{\mathrm{Exp}}}\in \left[0.93;1.31\right]\;eV$. This suggests that the parameterization of GGA functionals may introduce greater variability in $E_{g}$ predictions, even though the band profiles remain remarkably consistent, as indicated by the effective masses. The semi-local nature of GGA functionals appears to result in the estimation of higher $E_{g}$ relative to LDA. Notably, the GGA functionals that best align with the mean of the reported experimental $E_{g}$ range are BLYP $\left({\left[E_{g}\right]}_{\mathrm{BLYP}}^{{\mathrm{LCAO}}_{\mathrm{Exp}}}=1.26\;eV\right)$, KT2 $\left({\left[E_{g}\right]}_{\mathrm{KT}2}^{{\mathrm{LCAO}}_{\mathrm{Exp}}}=1.27\;eV\right)$, and XLYP $\left({\left[E_{g}\right]}_{\mathrm{XLYP}}^{{\mathrm{LCAO}}_{\mathrm{Exp}}}=1.31\;eV\right)$, which are typically employed in molecular system studies rather than solid-state ones. By contrast, PBEsol $\left({\left[E_{g}\right]}_{\mathrm{PBEsol}}^{{\mathrm{LCAO}}_{\mathrm{Exp}}}=1.01\;eV\right)$, a functional specifically optimized for solids, and the widely used PBE functional $\left({\left[E_{g}\right]}_{\mathrm{PBE}}^{{\mathrm{LCAO}}_{\mathrm{Exp}}}=1.11\;eV\right)$, known for its balanced description of molecular and solid-state systems, perform less favorably in this regard. This outcome indicates that accurately capturing the optoelectronic properties of CBS requires an effective Hamiltonian that achieves a delicate balance between local and nonlocal effects.

The partial density of states (PDoS) analysis for CBS, shown in Figures S1–S3, provides further insight into these results. Near the VBM, the density of states is predominantly composed of Cu and S orbitals, with negligible Bi contributions, indicating strong hybridization between Cu-3d and S-3p orbitals. Conversely, near the CBM, DoS is dominated by hybridized Bi-6p and S-3p orbitals, highlighting the importance of accurately capturing the localized covalent bonding within the CBS system. This may explain why functionals like BLYP, KT2, and XLYP perform better in predicting $E_{g}$, as they may better account for these bonding characteristics.

When considering higher-level functionals, such as meta-GGA and hybrid functionals (Figure 3), two surprising observations emerge. Both SCAN and R2SCAN meta-GGA functionals significantly underestimate $E_{g}$, predicting ${\left[E_{g}\right]}_{\mathrm{SCAN}}^{{\mathrm{LCAO}}_{\mathrm{Exp}}}=0.65\;eV$ and ${\left[E_{g}\right]}_{R2\mathrm{SCAN}}^{{\mathrm{LCAO}}_{\mathrm{Exp}}}=0.71\;eV$, lower than any LDA predictions. These functionals yield very compact valence bands, which may contribute to this underestimation. Conversely, the hybrid HSE06 functional, which is known to reliably estimate semiconductor bandgaps, overestimates considerably CBS’s $E_{g}$, predicting ${\left[E_{g}\right]}_{\mathrm{HSE}06}^{{\mathrm{LCAO}}_{\mathrm{Exp}}}=1.92\;eV$. This value far exceeds the upper limit of experimentally reported $E_{g}$, emphasizing that even higher-level functionals can struggle to provide reliable predictions for complex materials like CBS.

#### 3.1.2. PAW Calculations with Experimental Structure

The PAW method is a robust and versatile approach for solving the Kohn–Sham equations in DFT. By combining plane waves to describe valence electrons with augmentation functions to account for the effects of core electrons, PAW achieves near all-electron accuracy while maintaining computational efficiency. This dual treatment ensures accurate modeling of core-valence interactions, making PAW particularly effective for periodic systems like bulk crystals and materials containing heavier elements or localized d and f electrons.

Although PAW offers significant computational advantages over fully all-electron methods like LAPW, it is notably more resource-intensive than the LCAO method. This higher computational cost can be a limiting factor in studies requiring large supercells or extensive parameter exploration, such as those aimed at investigating how vacancies and dopants can influence charge carrier dynamics and enable bandgap engineering to optimize CBS as an absorber layer for photovoltaic applications. While the PAW method is generally expected to provide superior accuracy due to its near all-electron approach, this does not guarantee greater accuracy for the specific case of CBS.

**Figure 4:**
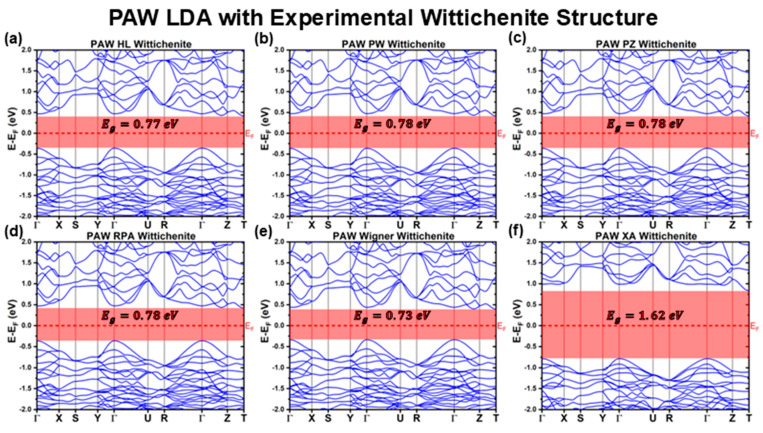
Cu_3_BiS_3_ band structures calculated using the PAW method for different LDA functionals, considering the experimental Wittichenite structure.

**Figure 5:**
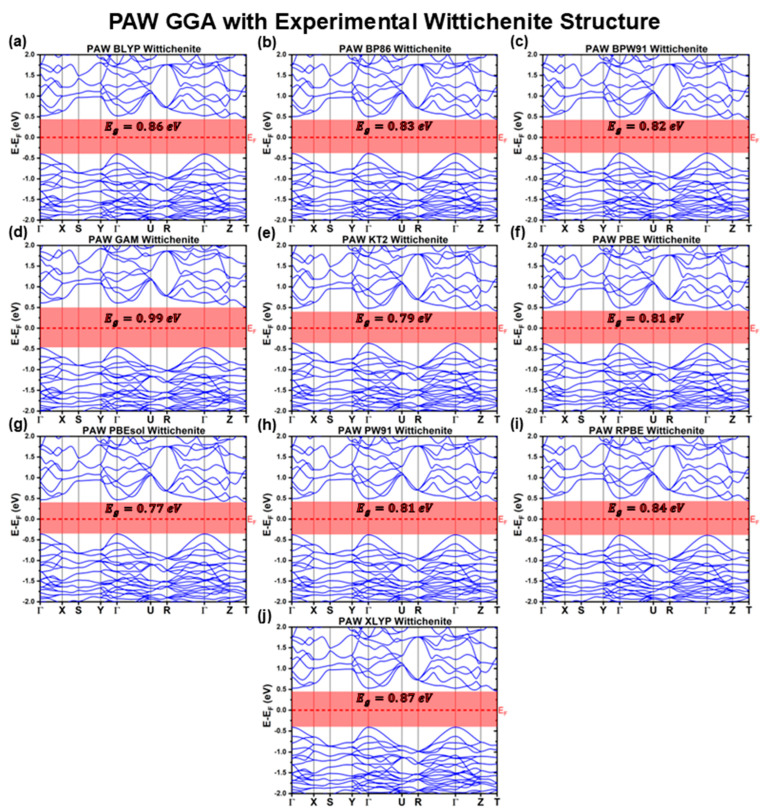
Cu_3_BiS_3_ band structures calculated using the PAW method for different GGA functionals, considering the experimental Wittichenite structure.

Figure 4 and Figure 5 illustrate the band structures of CBS calculated using the PAW method for the LDA and GGA XC functionals. From these band structures, the key optoelectronic properties were derived and comprehensively summarized in Table 3 and Table 4.

Similar to the LCAO method, the PAW calculations predict CBS to have an indirect bandgap, with the VBM located at the Γ point and the CBM at the T point of the Brillouin zone. Additionally, as observed with the LCAO method, the PAW results reveal a local minimum in the conduction band at the Γ point, with an energy close to that of the T point, resulting in a ${\left[E_{g}\right]}_{direct}$ comparable to the ${\left[E_{g}\right]}_{indirect}$.

For the PAW method using the LDA XC functional family, the band structures calculated with the HL, PW, PZ, RPA, and Wigner parameterizations are nearly identical, closely resembling each other and the band profiles of the GGA XC functionals. Furthermore, these band structures are remarkably similar to those obtained with the LDA and GGA functionals calculated using the LCAO method, except for the XA functional. This observation is reinforced by the effective mass calculations (Table 4), where values for both LDA and GGA functionals align with those from the PAW and LCAO methods. For instance, excluding the XA functional, the calculated effective masses are as follows:

- ${\left\{m_{h}^{*}\right\}}_{\mathrm{LDA}}^{{\mathrm{PAW}}_{\mathrm{Exp}}}={(-1.3\pm 0.1)m}_{0}={\left\{m_{h}^{*}\right\}}_{\mathrm{GGA}}^{{\mathrm{PAW}}_{\mathrm{Exp}}}\approx {\left\{m_{h}^{*}\right\}}_{\mathrm{LDA}}^{{\mathrm{LCAO}}_{\mathrm{Exp}}}={\left\{m_{h}^{*}\right\}}_{\mathrm{GGA}}^{{\mathrm{LCAO}}_{\mathrm{Exp}}}={(-1.4\pm 0.1)m}_{0}$, for the hole charge carriers along the $\Gamma_{X}$ direction.
- ${\left\{m_{h}^{*}\right\}}_{\mathrm{LDA}}^{{\mathrm{PAW}}_{\mathrm{Exp}}}={\left\{m_{h}^{*}\right\}}_{\mathrm{GGA}}^{{\mathrm{PAW}}_{\mathrm{Exp}}}={\left\{m_{h}^{*}\right\}}_{\mathrm{LDA}}^{{\mathrm{LCAO}}_{\mathrm{Exp}}}={\left\{m_{h}^{*}\right\}}_{\mathrm{GGA}}^{{\mathrm{LCAO}}_{\mathrm{Exp}}}={(-0.4\pm 0.1)m}_{0}$, for the hole charge carriers along the $\Gamma_{Y}$ direction.
- ${\left\{m_{h}^{*}\right\}}_{\mathrm{GGA}}^{{\mathrm{PAW}}_{\mathrm{Exp}}}={(-1.5\pm 0.1)m}_{0}\approx {\left\{m_{h}^{*}\right\}}_{\mathrm{LDA}}^{{\mathrm{PAW}}_{\mathrm{Exp}}}={\left\{m_{h}^{*}\right\}}_{\mathrm{LDA}}^{{\mathrm{LCAO}}_{\mathrm{Exp}}}={\left\{m_{h}^{*}\right\}}_{\mathrm{GGA}}^{{\mathrm{LCAO}}_{\mathrm{Exp}}}={(-1.4\pm 0.1)m}_{0}$, for the hole charge carriers along the $\Gamma_{Z}$ direction.
- ${\left\{m_{e}^{*}\right\}}_{\mathrm{LDA}}^{{\mathrm{PAW}}_{\mathrm{Exp}}}={\left\{m_{e}^{*}\right\}}_{\mathrm{GGA}}^{{\mathrm{PAW}}_{\mathrm{Exp}}}={\left\{m_{e}^{*}\right\}}_{\mathrm{LDA}}^{{\mathrm{LCAO}}_{\mathrm{Exp}}}={\left\{m_{e}^{*}\right\}}_{\mathrm{GGA}}^{{\mathrm{LCAO}}_{\mathrm{Exp}}}={(0.6\pm 0.1)m}_{0}$, for the electron charge carriers.

These results highlight the consistency of the effective mass predictions between the two methods and XC functional families. Both LDA and GGA functionals reveal an anisotropic nature for the hole effective masses, with significantly lower values along the $\Gamma_{Y}$ direction compared to the $\Gamma_{X}$ and $\Gamma_{Z}$ directions. A slight difference in the effective masses along $\Gamma_{X}$ and $\Gamma_{Z}$ was observed in the PAW calculations, with $\Gamma_{X}$ showing approximately ${(-1.3\pm 0.1)m}_{0}$ and $\Gamma_{Z}$ yielding ${(-1.4\pm 0.1)m}_{0}$ for LDA and ${(-1.5\pm 0.1)m}_{0}$ for GGA. However, this difference is minimal and can be considered negligible for practical purposes.

On the other hand, the XA functional exhibits a significantly different band profile compared to the other LDA and GGA functionals calculated using PAW. Notably, the XA band structure obtained with PAW closely resembles the XA band structure profile calculated using the LCAO method, a similarity further corroborated by their effective masses as follows:

- ${\left\{{\left(m_{h}^{*}\right)}_{\Gamma_{X}}\right\}}_{\mathrm{XA}}^{{\mathrm{PAW}}_{\mathrm{Exp}}}={\left\{{\left(m_{h}^{*}\right)}_{\Gamma_{X}}\right\}}_{\mathrm{XA}}^{{\mathrm{LCAO}}_{\mathrm{Exp}}}={-1.6m}_{0}$
- ${\left\{{\left(m_{h}^{*}\right)}_{\Gamma_{Y}}\right\}}_{\mathrm{XA}}^{{\mathrm{PAW}}_{\mathrm{Exp}}}=-0.6m_{0}\approx {\left\{{\left(m_{h}^{*}\right)}_{\Gamma_{Y}}\right\}}_{\mathrm{XA}}^{{\mathrm{LCAO}}_{\mathrm{Exp}}}=-0.8m_{0}$
- ${\left\{{\left(m_{h}^{*}\right)}_{\Gamma_{Z}}\right\}}_{\mathrm{XA}}^{{\mathrm{PAW}}_{\mathrm{Exp}}}=-2.0m_{0}\approx {\left\{{\left(m_{h}^{*}\right)}_{\Gamma_{Z}}\right\}}_{\mathrm{XA}}^{{\mathrm{LCAO}}_{\mathrm{Exp}}}=-1.9m_{0}$
- ${\left\{m_{e}^{*}\right\}}_{\mathrm{XA}}^{{\mathrm{PAW}}_{\mathrm{Exp}}}={\left\{m_{e}^{*}\right\}}_{\mathrm{XA}}^{{\mathrm{LCAO}}_{\mathrm{Exp}}}={0.6m}_{0}$.

Despite the similar band profiles, the predicted bandgap varies significantly between the PAW and LCAO methods. For all LDA functionals calculated using the PAW method, excluding the XA functional, the predicted ${\left[E_{g}\right]}_{\mathrm{indirect}}$ values are even lower than those obtained using LCAO. Specifically, the HL, PW, PZ, and RPA parameterizations yield $E_{g}\approx \left(0.78\pm 0.01\right)\;eV$, while the more rudimentary Wigner functional predicts an even lower value of ${\left[E_{g}\right]}_{\mathrm{Wigner}}^{{\mathrm{PAW}}_{\mathrm{Exp}}}=0.73\;eV$ (Table 3). Conversely, the XA functional exhibits the opposite trend in PAW calculations, with its bandgap increasing significantly from ${\left[E_{g}\right]}_{\mathrm{XA}}^{{\mathrm{LCAO}}_{\mathrm{Exp}}}=0.81\;eV$ in LCAO to ${\left[E_{g}\right]}_{\mathrm{XA}}^{{\mathrm{PAW}}_{\mathrm{Exp}}}=1.62\;eV$ in PAW. This value is more than double that predicted by the other PAW-based LDA functionals and slightly below the upper bound of the experimentally reported range (1–1.8 eV) [21,22,23,24,25,26,27,28,29,30,31,32].

For PAW calculations using GGA functionals, the predicted $E_{g}$ values are consistently higher than those obtained with LDA functionals (again, excluding XA). However, most fall within the narrow range of 0.77–0.86 eV, which is lower than the LCAO LDA results and, by extension, the LCAO GGA values. Interestingly, the GAM functional deviates from this trend, yielding ${\left[E_{g}\right]}_{\mathrm{GAM}}^{{\mathrm{PAW}}_{\mathrm{Exp}}}=0.99\;eV$ in PAW calculations, slightly exceeding all LCAO LDA values and surpassing the LCAO GAM functional (${\left[E_{g}\right]}_{\mathrm{GAM}}^{{\mathrm{LCAO}}_{\mathrm{Exp}}}=0.93\;eV$), which was the only LCAO GGA functional predicting a bandgap below 1 eV. The variability observed in the GAM functional likely stems from its design, which is optimized for homogeneous catalysis and contains a strong empirical component. The trade-offs inherent in its design are particularly detrimental to solid-state systems, leading to significant discrepancies, especially across different computational methods. Additionally, while the BLYP and XLYP functionals remain among the PAW GGA functionals predicting the highest bandgaps (${\left[E_{g}\right]}_{\mathrm{BLYP}}^{{\mathrm{PAW}}_{\mathrm{Exp}}}=0.86\;eV$ and ${\left[E_{g}\right]}_{\mathrm{XLYP}}^{{\mathrm{PAW}}_{\mathrm{Exp}}}=0.87\;eV$), the KT2 functional now predicts one of the lowest $E_{g}$ values (${\left[E_{g}\right]}_{\mathrm{KT}2}^{{\mathrm{PAW}}_{\mathrm{Exp}}}=0.79\;eV$). This likely arises from the KT2 functional’s niche optimization for improved magnetic resonance shielding, which may not translate well to solid-state electronic structure calculations.

Overall, these results indicate that, for CBS, the PAW method tends to underestimate the bandgap more severely than the LCAO method. Such an underestimation aligns with earlier CBS bandgap predictions, even when using more advanced functionals such as the hybrid HSE06 [61]. Moreover, this trend is also consistent with prior calculations employing Full-Potential LAPW with PBE functionals and/or Tran–Blaha modified Becke–Johnson (TB-mBJ) potentials, as reported in the works of Raju et al. and Oubakalla et al., where CBS bandgaps well below 1 eV were obtained [64,65].

The PDoS analysis from PAW calculations (Figures S4 and S5) qualitatively confirms the trends observed in the PDoS calculated using LCAO. The valence band remains predominantly composed of Cu and S states, while the conduction band is dominated by Bi and S states. However, in the PAW case, a notable reduction in Cu states is observed near the VBM (down to approximately $E-E_{F}=-1.5\;eV$), suggesting a weaker hybridization between Cu-3d and S-3p orbitals compared to LCAO calculations. This could further contribute to the discrepancies in the predicted bandgap values between the two methods.

### 3.2. Fully Relaxed Structures

While fixing the unit cell to an experimentally determined structure allows for a controlled assessment of how XC functionals and DFT methodologies influence the predicted optoelectronic properties of the intrinsic CBS, studying the effects of dopants and vacancies on CBS’s optoelectronic properties requires a different approach. Experimental lattice structures, typically obtained from bulk materials via X-ray diffraction or Transmission Electron Microscopy, do not accurately capture the localized distortions induced by dilute dopants or intrinsic defects. Even at doping concentrations of around 100 ppm, which is exceptionally high by semiconductor bandgap engineering standards, these perturbations have a negligible impact on the overall bulk crystal structure.

Consequently, DFT studies investigating the role of defects and dopants must allow for full structural relaxation of the modified supercell to obtain an optimized equilibrium configuration. This requires identifying which DFT methods and XC functionals can most accurately predict CBS’s structural properties. To this end, full structure optimizations were performed, starting from the experimental reference unit cell reported by Kocman et al. [95]. This structure was selected for its highly reliable Powder Diffraction File (PDF), as indicated by its high score in the International Centre for Diffraction Data (ICDD) database.

Table 5 summarizes the lattice parameters of several experimentally reported CBS Wittichenite (*P*2_1_2_1_2_1_ space group) unit cells, including the reference structure used in this study. The comparison of these experimental datasets highlights the variability in reported lattice parameters, providing a benchmark to assess the accuracy of DFT-optimized structures.

As observed in Table 5, the reported experimental lattice parameters for CBS exhibit variations within a tolerance ≲1%. Given this inherent variability even among experimental structures, expecting DFT-predicted structures to match the experimental reference with deviations smaller than 1% is unrealistic. However, this range serves as a meaningful reference to evaluate the reliability of the structural predictions obtained from different DFT approaches.

#### 3.2.1. LCAO Calculations with Fully Relaxed Structure

Table 6 provides insight into the accuracy of different functionals within the LCAO method to predict the optimized CBS lattice parameters. Once again, the XA functional deviates significantly from all other LDA functionals, producing the most inaccurate structural parameters across all computed functionals, including those from the GGA, meta-GGA, and hybrid families. The XA functional underestimates the a lattice parameter by a striking 19.5% and exhibits an average absolute relative deviation $\left(\langle ∆\rangle =\frac{\left|∆a\right|+\left|∆b\right|+\left|∆c\right|}{3}\right)$ of 14.3%. When excluding the XA functional, the remaining LDA functionals display a more consistent trend, generally underestimating a by approximately 5% and c by around 8%, while slightly overestimating b.

A systematic evaluation focusing on $\langle ∆\rangle$ across all the LDA functionals, excluding XA, leads to conclude that the LDA functionals exhibit a mean $\langle ∆\rangle$ deviation of ${\bar{\langle ∆\rangle }}_{\mathrm{LDA}}^{{\mathrm{LCAO}}_{\mathrm{DFT}}}=4.6\%$ with a remarkably low standard deviation of ${\left[\sigma_{\bar{\langle ∆\rangle }}\right]}_{\mathrm{LDA}}^{{\mathrm{LCAO}}_{\mathrm{DFT}}}={\left[\sqrt{\frac{1}{N}\sum_{i}^{N}{\left({\bar{∆}}_{i}-\langle \bar{∆}\rangle \right)}^{2}}\right]}_{\mathrm{LDA}}^{{\mathrm{LCAO}}_{\mathrm{DFT}}}=0.3\%$, indicating a uniform performance across this family. Interestingly, the simplest LDA functional, Wigner, provides the closest agreement to experimental lattice parameters, with ${\langle ∆\rangle }_{\mathrm{Wigner}}^{{\mathrm{LCAO}}_{\mathrm{DFT}}}=4.2\%$, although this deviation still exceeds considerably the experimental variability observed in Table 5.

For the GGA functionals computed within the LCAO method, most functionals yield improved agreement with experimental structures when compared to the LDA family, resulting in a lower ${\bar{\langle ∆\rangle }}_{\mathrm{GGA}}^{{\mathrm{LCAO}}_{\mathrm{DFT}}}=3.5\%$. However, the variability among individual functionals is significantly larger, spanning the range ${\langle ∆\rangle }_{\mathrm{LDA}}^{{\mathrm{LCAO}}_{\mathrm{DFT}}}\in [1.2\%;5.1\%]$ and a ${\left[\sigma_{\bar{\langle ∆\rangle }}\right]}_{\mathrm{GGA}}^{{\mathrm{LCAO}}_{\mathrm{DFT}}}=1.3\%$. Additionally, convergence issues were encountered for the BPW91 and GAM functionals, preventing the Hellmann–Feynman forces from converging below $0.01\;\mathrm{eV}{Å}^{-1}$. Despite thousands of geometry optimization cycles (each with fully converged SCF calculations) the final residual forces remained at 0.17 $\mathrm{eV}{Å}^{-1}$ and 0.12 $\mathrm{eV}{Å}^{-1}$, respectively. Consequently, their results should be interpreted with caution.

Among the GGA functionals, several exhibit excellent agreement with experimental values, notably PW91, BP86, BPW91 (despite its convergence issues), and RPBE, which yield $\langle ∆\rangle$ values of ${\langle ∆\rangle }_{\mathrm{PW}91}^{{\mathrm{LCAO}}_{\mathrm{DFT}}}=1.2\%$, ${\langle ∆\rangle }_{\mathrm{BP}86}^{{\mathrm{LCAO}}_{\mathrm{DFT}}}=2.1\%$, ${\langle ∆\rangle }_{\mathrm{BPW}91}^{{\mathrm{LCAO}}_{\mathrm{DFT}}}=2.3\%$ and ${\langle ∆\rangle }_{\mathrm{RPBE}}^{{\mathrm{LCAO}}_{\mathrm{DFT}}}=2.5\%$, respectively. These deviations are relatively small, with PW91 being especially close to the range of reported experimental variability, making it a particularly promising functional for CBS structure optimizations, especially considering its low computational cost (Table S1 and Figures S11 and S12). Interestingly, the PBEsol XC functional, which was specifically designed to improve the prediction of structural properties in solid-state bulk materials, performs relatively poorly in predicting the CBS lattice parameters within the LCAO framework. Similarly, the widely used and theoretically well-balanced PBE functional, often regarded as a versatile choice for both molecular and solid-state systems, yields an average absolute relative deviation of ${\langle ∆\rangle }_{\mathrm{PBE}}^{{\mathrm{LCAO}}_{\mathrm{DFT}}}=3.7\%$, just above the GGA mean of ${\bar{\langle ∆\rangle }}_{\mathrm{GGA}}^{{\mathrm{LCAO}}_{\mathrm{DFT}}}=3.5\%$. These findings reinforce the necessity of systematically assessing the most suitable XC functional for describing specific material properties rather than assuming that commonly used functionals will always provide the most accurate results for a given class of materials.

For the meta-GGA functionals, both SCAN and R2SCAN show relatively good performance (particularly R2SCAN), with ${\langle ∆\rangle }_{\mathrm{SCAN}}^{\mathrm{LCAO}}=3.1\%$ and ${\langle ∆\rangle }_{R2\mathrm{SCAN}}^{\mathrm{LCAO}}=1.8\%$, respectively. However, their performance is comparable to, or in some cases worse than, many GGA functionals; hence, the added computational cost of meta-GGA does not offer a clear advantage for CBS structural optimization.

Finally, the hybrid HSE06 functional provides the most accurate structural predictions, with ${\langle ∆\rangle }_{\mathrm{HSE}06}^{{\mathrm{LCAO}}_{\mathrm{DFT}}}=0.9\%$, falling below the 1% mark and closely matching experimental variability. However, despite its accuracy, the substantial computational cost of HSE06 makes it a less practical choice for large-scale simulations, especially when the computationally inexpensive PW91 functional (Table S1 and Figures S11 and S12) provides comparably accurate results.

**Figure 6:**
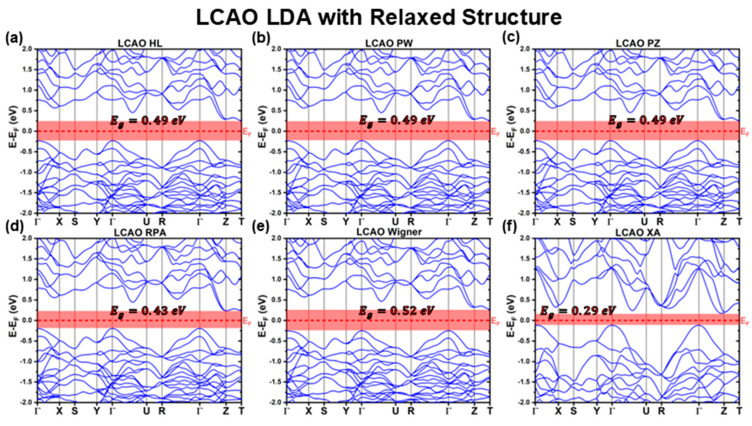
Cu_3_BiS_3_ band structures calculated using the LCAO method for different LDA functionals, considering relaxed structures.

**Figure 7:**
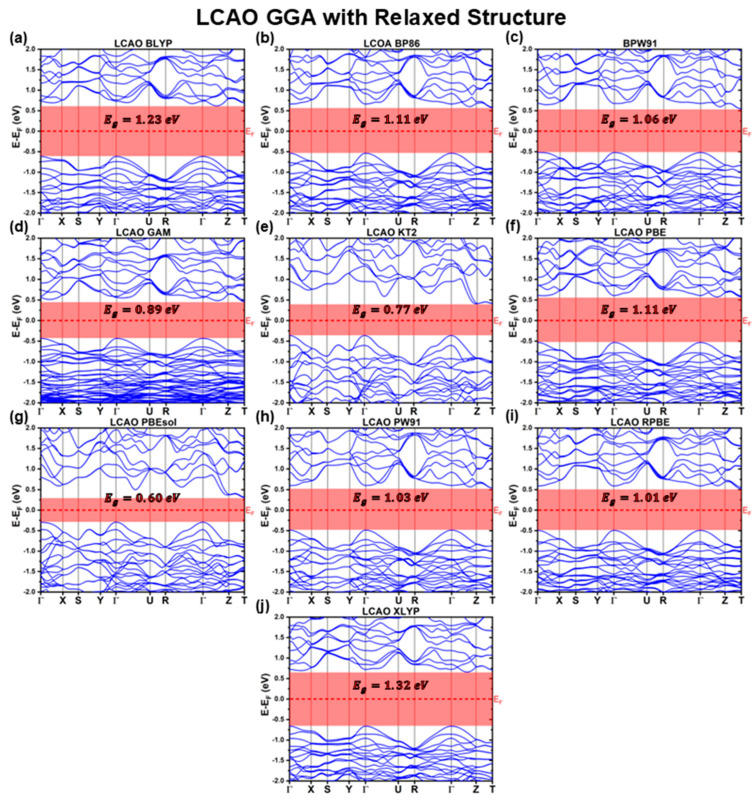
Cu_3_BiS_3_ band structures calculated using the LCAO method for different GGA functionals, considering relaxed structures.

**Figure 8:**
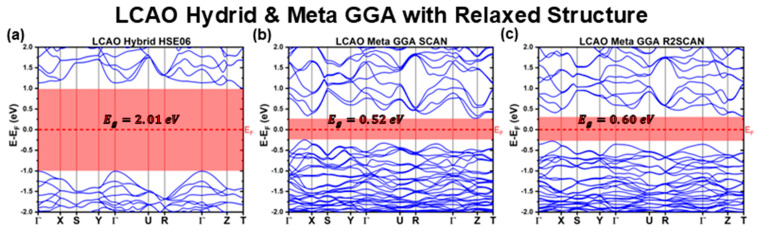
Cu_3_BiS_3_ band structures calculated using the LCAO method for different meta-GGA and Hybrid functionals, considering relaxed structures.

Figure 6, Figure 7 and Figure 8 present the band structures calculated using the LCAO method for the fully relaxed structures obtained with different XC functionals. The key optoelectronic properties extracted from these band structures are summarized in Table 7 and Table 8.

Consistent with the previous calculations using the experimental reference structure, all relaxed CBS band structures retain an indirect bandgap, with the VBM located at the Γ point. However, the CBM is not always located at the T point. Specifically, for the RPA functional, the CBM shifts to the Z point, while for the XA, BLYP, KT2, XLYP, and SCAN functionals, it is positioned along the Γ−Z path, as detailed in Table 8.

After structural relaxation, the band profiles of the LDA functionals, shown in Figure 6, exhibit substantial deviations from those obtained using the fixed experimental reference structure (Figure 1). Among the LDA functionals, all display remarkably similar band structures, except for the XA functional, which deviates significantly from both the other relaxed LDA band structures and its own LDA counterpart computed using the experimental structure. These structural modifications lead to a substantial reduction in the predicted bandgap, as shown in Table 7. The LDA bandgaps decrease from ${\left[E_{g}\right]}_{\mathrm{LDA}}^{{\mathrm{LCAO}}_{\mathrm{Exp}}}\approx 0.93\;eV$ (using the experimental reference structure) to ${\left[E_{g}\right]}_{\mathrm{HL}}^{{\mathrm{LCAO}}_{\mathrm{DFT}}}={\left[E_{g}\right]}_{\mathrm{PW}}^{{\mathrm{LCAO}}_{\mathrm{DFT}}}={\left[E_{g}\right]}_{\mathrm{PZ}}^{{\mathrm{LCAO}}_{\mathrm{DFT}}}=0.49\;eV$ for the HL, PW, and PZ functionals, ${\left[E_{g}\right]}_{\mathrm{Wigner}}^{{\mathrm{LCAO}}_{\mathrm{DFT}}}=0.52\;eV$ for Wigner, ${\left[E_{g}\right]}_{\mathrm{RPA}}^{{\mathrm{LCAO}}_{\mathrm{DFT}}}=0.43\;eV$ for RPA, and as low as ${\left[E_{g}\right]}_{\mathrm{XA}}^{{\mathrm{LCAO}}_{\mathrm{DFT}}}=0.29\;eV$ for XA.

The slight variations in $E_{g}$ for the RPA and Wigner functionals stem from differences in their band structures, particularly along the $\Gamma_{X}$ direction and, in the case of RPA, at the Z symmetry point, which is now its new CBM. These modifications are also reflected in the effective mass values reported in Table 8.

While the anisotropic nature of the hole effective masses is preserved, structural relaxation leads to a consistent reduction in ${\left(m_{h}^{*}\right)}_{\Gamma_{Z}}$ to approximately ${(-0.8\pm 0.1)m}_{0}$ for all LDA functionals, including XA. Meanwhile, ${\left(m_{h}^{*}\right)}_{\Gamma_{Y}}$ remains at $=-0.5m_{0}$ for all LDA functionals except XA. For ${\left(m_{h}^{*}\right)}_{\Gamma_{X}}$, there is a systematic increase compared to the calculations with the fixed experimental structure, with values ranging from ${\left\{{\left(m_{h}^{*}\right)}_{\Gamma_{X}}\right\}}_{\mathrm{Wigner}}^{{\mathrm{LCAO}}_{\mathrm{DFT}}}=-1.5m_{0}$ to ${\left\{{\left(m_{h}^{*}\right)}_{\Gamma_{X}}\right\}}_{\mathrm{HL}}^{{\mathrm{LCAO}}_{\mathrm{DFT}}}={\left\{{\left(m_{h}^{*}\right)}_{\Gamma_{X}}\right\}}_{\mathrm{PW}}^{{\mathrm{LCAO}}_{\mathrm{DFT}}}={\left\{{\left(m_{h}^{*}\right)}_{\Gamma_{X}}\right\}}_{\mathrm{PZ}}^{{\mathrm{LCAO}}_{\mathrm{DFT}}}=-2.0m_{0}$, and ${\left\{{\left(m_{h}^{*}\right)}_{\Gamma_{X}}\right\}}_{\mathrm{RPA}}^{{\mathrm{LCAO}}_{\mathrm{DFT}}}=-2.5m_{0}$. The XA functional, as a consistent outlier, predicts an overwhelming ${\left\{{\left(m_{h}^{*}\right)}_{\Gamma_{X}}\right\}}_{\mathrm{XA}}^{{\mathrm{LCAO}}_{\mathrm{DFT}}}=-15.9m_{0}$, due to its unusually flat valence band at $\Gamma_{X}$.

Regarding electron effective masses, the HL, PW, PZ, and Wigner functionals, which retain the CBM at T, predict ${\left(m_{e}^{*}\right)}_{T}=0.6m_{0}$. However, for RPA and XA, which now have their CBM at Z and along the Γ−Z path, respectively, the electron effective masses decrease to $0.4m_{0}$.

For the GGA functional family, the band structures of most relaxed CBS structures closely resemble their constrained experimental counterparts, with both the VBM and CBM retaining the same symmetry points. However, two notable exceptions are the KT2 and PBEsol functionals, which predict band structures more similar to the LDA-relaxed results (excluding XA). The KT2 functional exhibits additional band distortions, shifting the CBM to approximately 910 along the Γ−Z path.

Further deviations are observed for BPW91, GAM, BLYP, and XLYP functionals. While their band structures remain qualitatively similar to the rest of the GGA functionals, they exhibit distinct distortions. For BLYP and XLYP, the CBM shifts to a symmetry point along the Γ−Z path, GAM displays a compressed valence band, and BPW91 shows significant band flattening at the Γ point along the $\Gamma_{X}$ direction. These distortions lead to variations in the predicted effective masses. The KT2 functional results in lower ${\left(m_{h}^{*}\right)}_{\Gamma_{X}}$ and ${\left(m_{h}^{*}\right)}_{\Gamma_{Z}}$, while the GAM functional predicts increased hole effective masses along these directions. Additionally, BLYP and XLYP show an increase in ${\left(m_{h}^{*}\right)}_{\Gamma_{Z}}$. However, the most pronounced deviations occur for PBEsol and BPW91. The PBEsol functional predicts nearly double the ${\left(m_{h}^{*}\right)}_{\Gamma_{X}}$ value but half the ${\left(m_{h}^{*}\right)}_{\Gamma_{Z}}$ compared to its experimental structure counterpart. Meanwhile, BPW91 retains similar effective masses in most directions but exhibits an extreme increase at $\Gamma_{X}$, with ${\left\{{\left(m_{h}^{*}\right)}_{\Gamma_{X}}\right\}}_{\mathrm{BPW}91}^{{\mathrm{LCAO}}_{\mathrm{DFT}}}=-22.9m_{0}$, due to substantial band flattening.

Despite these band structure distortions, the majority of GGA functionals predict bandgap values that remain relatively consistent with their calculations considering the experimental reference structure. BLYP, BP86, BPW91, GAM, PBE, and XLYP functionals show variations of less than 5% (under 60 meV), while PW91 and RPBE differ by about 8% (around 90 meV). The KT2 and PBEsol functionals, however, exhibit significant deviations of approximately 40% (500 and 400 meV, respectively). Notably, all functionals underestimate $E_{g}$ relative to their experimental reference structures, except for XLYP, which slightly overestimates $E_{g}$ by just 10 meV. This overall consistency in optoelectronic properties is partly attributed to the relatively accurate lattice parameter predictions for most GGA functionals.

While the CBS structures optimized using meta-GGA functionals also exhibit minimal deviations in lattice parameters compared to the experimental reference, their band structures (Figure 8) display significant distortions. This suggests that, although the lattice constants remain similar, the relative atomic positions undergo substantial rearrangements. As a result, the VBM shifts away from the Γ point, moving along the Γ−X path. Both SCAN and R2SCAN functionals further underestimate $E_{g}$, with reductions of approximately 19% and 15%, respectively, compared to their already underestimated experimental reference calculations. These findings reinforce the conclusion that SCAN and R2SCAN, when computed using the LCAO formalism, are ill-suited to predict both the structural and optoelectronic properties of CBS.

In contrast, the relaxed HSE06 hybrid functional retains nearly all features of its experimental reference structure counterpart, yielding almost identical band structures and effective masses. The bandgap remains consistent at ${\left[E_{g}\right]}_{\mathrm{HSE}06}^{{\mathrm{LCAO}}_{\mathrm{DFT}}}=2.01\;eV$*,* compared to ${\left[E_{g}\right]}_{\mathrm{HSE}06}^{{\mathrm{LCAO}}_{\mathrm{Exp}}}=1.92\;eV$, representing only a 5% deviation. Additionally, the charge carrier effective masses remain largely unchanged, with ${\left(m_{h}^{*}\right)}_{\Gamma_{Y}}$, ${\left(m_{h}^{*}\right)}_{\Gamma_{Z}}$, and $m_{e}^{*}$ being nearly identical to their experimental structure counterparts. The only notable deviation is a slight band flattening at the Γ point along the $\Gamma_{X}$ direction, increasing the absolute value of ${\left(m_{h}^{*}\right)}_{\Gamma_{X}}$ from $1.3m_{0}$ to $1.9m_{0}$. This consistency aligns with the minimal differences between the experimental reference lattice parameters and the fully relaxed HSE06 structure.

The PDoS for the LCAO-relaxed structures are presented in Figures S6–S8. While each functional family (and individual XC functional) exhibits slight quantitative variations, such as compression or stretching of the density of states along both the energy (x) and state density (y) axes, the overall qualitative characteristics remain consistent with those obtained from the LCAO calculations considering the experimental reference structure. In all cases, the valence band remains predominantly composed of Cu and S states, whereas the conduction band is primarily dominated by Bi and S states.

#### 3.2.2. PAW Calculations with Fully Relaxed Structure

Finally, Table 9 provides a comparative assessment of the accuracy of different functionals within the PAW method to predict the optimized CBS lattice parameters. A cross-analysis of Table 6 and Table 9 reveals that the structural optimization trends observed for the LDA and GGA functional families remain consistent across both the LCAO and PAW methods. However, the PAW method demonstrates a slight overall improvement in lattice parameter predictions compared to the LCAO.

For the LDA functional family, the XA functional remains a clear outlier, yielding approximately the same $\langle ∆\rangle$ value as in the LCAO calculations. Excluding XA, the LDA functionals computed using the PAW method exhibit a ${\bar{\langle ∆\rangle }}_{\mathrm{LDA}}^{{\mathrm{PAW}}_{\mathrm{DFT}}}=4.2\%$, with a standard deviation of ${\left[\sigma_{\bar{\langle ∆\rangle }}\right]}_{\mathrm{LDA}}^{{\mathrm{PAW}}_{\mathrm{DFT}}}=0.4\%$.These values are nearly identical to those obtained using the LCAO method. As before, the HL, PW, and PZ functionals predict nearly the same $\langle ∆\rangle$ values, while the Wigner and RPA functionals display the lowest and highest deviations, respectively, now with ${\langle ∆\rangle }_{\mathrm{Wigner}}^{{\mathrm{PAW}}_{\mathrm{DFT}}}=3.6\%$ and ${\langle ∆\rangle }_{\mathrm{RPA}}^{{\mathrm{PAW}}_{\mathrm{DFT}}}=4.9\%$.

Similarly, for the GGA family, the mean of the average deviation improves to ${\bar{\langle ∆\rangle }}_{\mathrm{GGA}}^{{\mathrm{PAW}}_{\mathrm{DFT}}}=2.8\%$, though with a slightly wider range of $\langle ∆\rangle$ values spanning ${\langle ∆\rangle }_{\mathrm{LDA}}^{{\mathrm{PAW}}_{\mathrm{DFT}}}\in [1.2\%;5.2\%]$, resulting in a standard deviation of ${\left[\sigma_{\bar{\langle ∆\rangle }}\right]}_{\mathrm{GGA}}^{{\mathrm{PAW}}_{\mathrm{DFT}}}=1.5\%$. The PW91, BP86, BPW91, and RPBE functionals continue to provide the best agreement with experimental lattice parameters, yielding deviations of ${\langle ∆\rangle }_{\mathrm{PW}91}^{{\mathrm{PAW}}_{\mathrm{DFT}}}=1.3\%$, ${\langle ∆\rangle }_{\mathrm{BP}86}^{{\mathrm{PAW}}_{\mathrm{DFT}}}=1.8\%$, ${\langle ∆\rangle }_{\mathrm{BPW}91}^{{\mathrm{PAW}}_{\mathrm{DFT}}}=1.2\%$ and ${\langle ∆\rangle }_{\mathrm{RPBE}}^{{\mathrm{PAW}}_{\mathrm{DFT}}}=1.4\%$, all below 2%. Moreover, the PBE functional shows an improvement in lattice parameter predictions when using the PAW method, with ${\langle ∆\rangle }_{\mathrm{PBE}}^{{\mathrm{PAW}}_{\mathrm{DFT}}}=1.4\%$, performing considerably better than its LCAO counterpart. On the other hand, the PBEsol functional remains one of the least accurate for CBS lattice parameter predictions, yielding ${\langle ∆\rangle }_{\mathrm{PBEsol}}^{{\mathrm{PAW}}_{\mathrm{DFT}}}=4.0\%$, identical to its LCAO result.

Overall, despite some minor improvements for certain functionals, the conclusions regarding lattice parameter optimization using LDA and GGA functionals remain nearly identical for both the LCAO and PAW methods, reinforcing the reliability of the observed trends.

**Figure 9:**
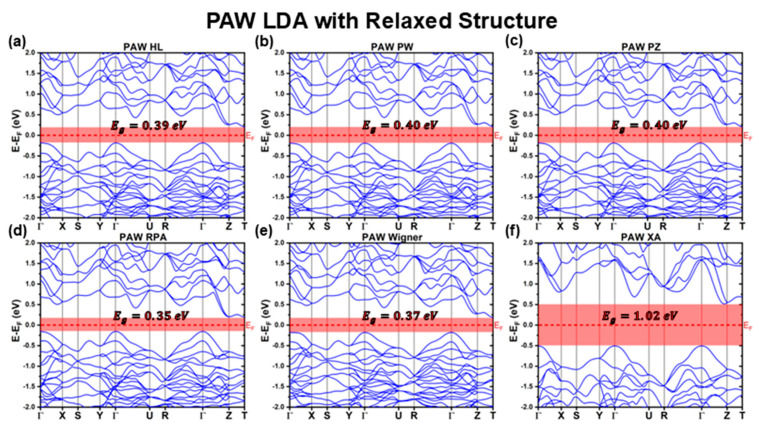
Cu_3_BiS_3_ band structures calculated using the PAW method for different LDA functionals, considering relaxed structures.

**Figure 10:**
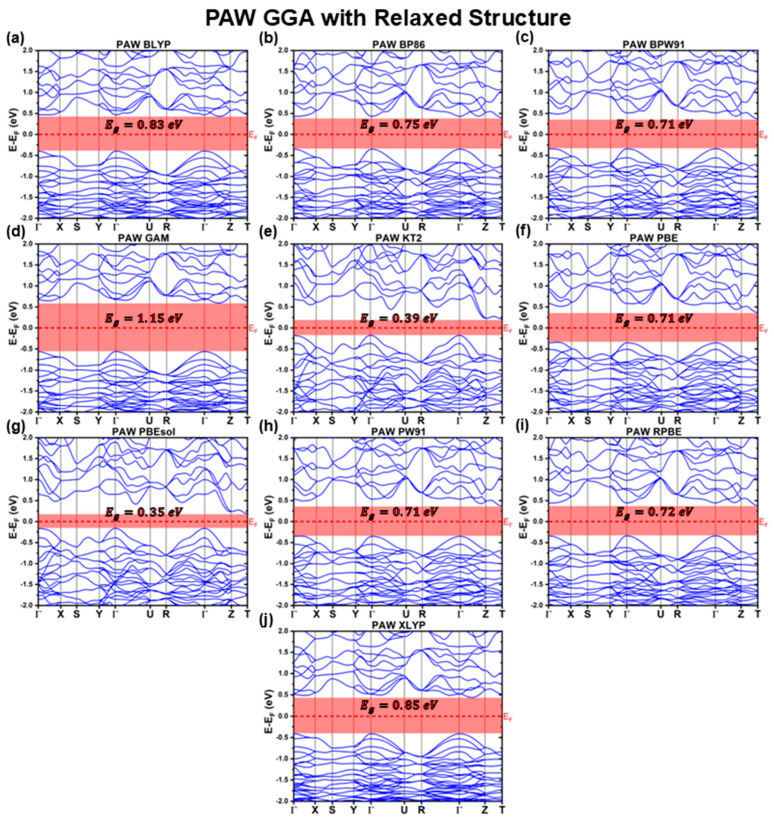
Cu_3_BiS_3_ band structures calculated using the PAW method for different GGA functionals, considering relaxed structures.

Figure 9 and Figure 10 present the band structures obtained using the PAW method for the fully relaxed structures, considering different XC functionals. The key optoelectronic properties extracted from these calculations are summarized in Table 10 and Table 11.

After structural optimization, the band structure profiles of the LDA XC functional family calculated using PAW closely resemble those obtained with the LCAO method, showing near one-to-one equivalence. However, an evident distinction is the flattening of the along the $\Gamma_{X}$ direction for all functionals except XA. This band flattening leads to an appreciable increase in the ${\left(m_{h}^{*}\right)}_{\Gamma_{X}}$, while the remaining effective masses remain mostly unchanged. The XA functional, which already exhibited anomalous behavior in the relaxed LCAO calculations, continues to deviate from the other LDA functionals. Although its band structure presents a similar profile to its LCAO counterpart, noticeable differences in band curvature at the VBM lead to distinct hole effective masses (Table 11). Additionally, vertical shifts between individual bands result in a significant increase in its predicted bandgap, rising from ${\left[E_{g}\right]}_{\mathrm{XA}}^{{\mathrm{LCAO}}_{\mathrm{DFT}}}=0.29$ to ${\left[E_{g}\right]}_{\mathrm{XA}}^{{\mathrm{PAW}}_{\mathrm{DFT}}}=1.02\;\mathrm{eV}$ (Table 10).

For the remaining LDA functionals, structural relaxation leads to a further reduction in the bandgap, mirroring the trend observed in the relaxed LCAO results. As a consequence, the PAW-relaxed structures predict the lowest $E_{g}$ values among all LDA functionals in this study, with bandgaps ranging between 0.35 and 0.40 eV, less than half of the experimentally reported lower limit range of 1 eV.

In the case of GGA functionals, the PAW-relaxed structures exhibit band structure distortions analogous to those observed in the LCAO-relaxed calculations. These distortions lead to changes in the band edge positions relative to the PAW calculations performed using the experimental structure. Specifically, the VBM in the PBE and PW91 functionals shifts from the Γ point to an intermediate position along the Γ−X path, while the CBM in the GAM and KT2 functionals also changes, shifting from T to an intermediate point along Γ−Z for GAM and from an intermediate point along Γ−Z to T for KT2.

These changes in band structure result in significant variations in effective masses, particularly for ${\left(m_{h}^{*}\right)}_{\Gamma_{X}}$ and ${\left(m_{h}^{*}\right)}_{Z}$. The range of ${\left\{{\left(m_{h}^{*}\right)}_{\Gamma_{X}}\right\}}_{\mathrm{GGA}}^{{\mathrm{PAW}}_{\mathrm{DFT}}}$ spans from $-1.4\;m_{0}\;\mathrm{to}-19.9\;m_{0}$, with BPW91 yielding the largest absolute ${\left(m_{h}^{*}\right)}_{\Gamma_{X}}$ value. This result stems from the pronounced flattening of the BPW91 VBM along $\Gamma_{X}$ and is consistent with the previously observed ${\left\{{\left(m_{h}^{*}\right)}_{\Gamma_{X}}\right\}}_{\mathrm{BPW}91}^{{\mathrm{LCAO}}_{\mathrm{DFT}}}=-22.9m_{0}$. This confirms that the effect is intrinsic to the system relaxed using the BPW91 XC functional rather than an artifact caused by incomplete structural relaxation below the adopted tolerance threshold.

Regarding the ${\left\{m_{e}^{*}\right\}}_{\mathrm{GGA}}^{{\mathrm{PAW}}_{\mathrm{DFT}}}$ and ${\left\{{\left(m_{h}^{*}\right)}_{\Gamma_{Z}}\right\}}_{\mathrm{GGA}}^{{\mathrm{PAW}}_{\mathrm{DFT}}}$, while their values exhibit slightly greater variability in the PAW-relaxed GGA functionals compared to those computed using both LCAO and PAW considering the experimental reference structure, this variation remains significantly lower than that observed for ${\left\{{\left(m_{h}^{*}\right)}_{\Gamma_{X}}\right\}}_{\mathrm{GGA}}^{{\mathrm{PAW}}_{\mathrm{DFT}}}$. In contrast, ${\left\{{\left(m_{h}^{*}\right)}_{\Gamma_{Y}}\right\}}_{\mathrm{GGA}}^{{\mathrm{PAW}}_{\mathrm{DFT}}}$ consistently maintains a value of approximately $-0.5m_{0}$ across all GGA functionals, confirming that the $\Gamma_{Y}$ direction corresponds to the lowest absolute hole effective mass, as observed in all previously discussed cases.

As discussed in Section 3.1.2, the PAW method generally underestimates the CBS bandgap even more than the LCAO method. Consequently, following a similar trend to that observed for LCAO-relaxed structures, the PAW-relaxed GGA structures also predict lower $E_{g}$ values than their PAW experimental reference structure counterparts. The magnitude of this underestimation varies among functionals. The BLYP and XLYP functionals show the smallest deviation, around 3% (≈30 meV), while BP86 underestimates $E_{g}$ by about 10% (≈80 meV). PBE, PW91, and RPBE exhibit even greater underestimation, around 12−14% (≈110 meV). Yet, the most significant deviations are observed for KT2 and PBEsol, which underestimate $E_{g}$ by more than 50% (>400 meV).

Interestingly, while optimized structures for all functionals except XA underestimated $E_{g}$ in both LCAO and PAW calculations, the GAM functional is now an exception in the PAW-relaxed case, overestimating the bandgap by ≈16% (≈160 meV). As previously stated, this deviation is likely due to GAM’s highly empirical nature, which leads to inconsistencies when applied to different computational methodologies.

The PDoS analysis for the PAW-relaxed structures (Figures S9 and S10) qualitatively reinforces the trends observed in the LCAO-calculated PDoS for both the constrained and relaxed structures. As previously noted, the valence band remains primarily composed of Cu and S states, while the conduction band is dominated by Bi and S states. However, similar to the PAW calculations considering the experimental reference structure, a notable reduction in Cu states near the VBM is observed. This suggests a weaker hybridization between Cu-3d and S-3p orbitals in the PAW method as compared to LCAO. These differences highlight the intrinsic characteristics of each method and may partially explain the discrepancies observed in the predicted optoelectronic properties between PAW and LCAO.

Overall, while the PAW method provides a better description of CBS’s lattice parameters than LCAO, its deviations in optoelectronic properties are more pronounced, particularly after full structural relaxation. This discrepancy highlights the challenges of selecting an optimal computational approach for accurately describing CBS’s electronic structure.

Notably, the BLYP and XLYP GGA functionals consistently provided more accurate optoelectronic predictions, whereas meta-GGA functionals performed poorly. This can be attributed to the electronic structure of CBS, where the valence band primarily consists of hybridized Cu-3d and S-3p states, while the conduction band is dominated by Bi-6p and S-3p states. This intricate hybridization demands an XC functional capable of accurately describing both localized (Cu-3d) and delocalized (Bi-6p) electron densities. BLYP and XLYP effectively balance exchange and correlation effects, mitigating excessive delocalization or overbinding, resulting in bandgap predictions that align more closely with experimental values. Furthermore, given CBS’s pronounced covalent character, functionals originally developed for molecular systems, such as BLYP and XLYP, appear to better capture its band structure than solid-state functionals like PBEsol, which was specifically designed for densely packed solids. In contrast, meta-GGA functionals, which try to enhance conventional GGA by incorporating density-gradient and kinetic-energy-density terms, often excel in describing transition metal compounds and oxides but struggle with materials like CBS, where strong hybridization between localized and delocalized states plays a critical role.

Lastly, for nearly all XC functionals considered in this study, the calculated Fermi level does not lie exactly at the midpoint of the bandgap, regardless of whether the LCAO or PAW method is used or whether the experimental reference structure or the DFT-optimized structures are considered. Instead, it consistently shifts slightly toward the VBM (as illustrated in Table 1, Table 3, Table 7 and Table 10), with deviations reaching up to 29 meV, despite the calculations being performed under T=0 K conditions. This result suggests that CBS inherently exhibits p-type behavior, a desirable characteristic for absorber layers in TF PVs.

## 4. Conclusions

This study systematically examined the impact of DFT methodologies (LCAO and PAW) and XC functionals on the structural and optoelectronic properties of CBS, a promising earth-abundant absorber for thin-film photovoltaics. Understanding these computational influences is essential, particularly for future studies involving large supercells to investigate dopants, vacancies, and other defects for bandgap and charge carrier engineering.

Both LCAO and PAW consistently predicted an indirect bandgap for CBS across all XC functionals, aligning with most previous DFT studies [60,61,62,63,64,65]. However, this contradicts experimental reports, which often classify CBS as a direct bandgap material [28,29,30,31,32]. This discrepancy may stem, in part, from the first DFT study on CBS, which predicted a direct bandgap [59], potentially influencing how subsequent experimental data were interpreted. Experimental bandgaps are typically determined using Tauc-like plots, where the absorption coefficient α is plotted as a function of photon energy hν, with the ordinate given by ${\left(\alpha h\nu \right)}^{\beta }$. The exponent β depends on the assumed nature of the bandgap: β=2 for ${\left[E_{g}\right]}_{direct}$ and $\beta =\frac{1}{2}$ for ${\left[E_{g}\right]}_{indirect}$. Since real absorption spectra are not perfectly linear over the entire energy range, researchers must select a region that best fits a straight line, introducing a degree of subjectivity. Similarly, Whittles et al. [61] combined experimental optical data analysis with HSE06 + SOC calculations and concluded that CBS is a weakly indirect bandgap semiconductor, with absorption primarily occurring above 1.4 eV. They also identified multiple optoelectronic critical points within a narrow energy range, making conventional absorption analysis methods unreliable, as the bandgap cannot be accurately determined through simple linear extrapolation of ${\left(\alpha h\nu \right)}^{\beta }$ vs. hν plot for any assumed β-value. Furthermore, the small direct-indirect bandgap difference, observed here and in several DFT studies, worsens this ambiguity.

From the DFT calculations considering the experimental reference for the CBS structure, it is possible to conclude that the PAW method, while expected to be more accurate due to its near all-electron treatment, systematically underestimated the CBS bandgap even more than LCAO.

The LDA and meta-GGA XC functionals severely underestimate CBS’s band gap, for both the LCAO and PAW methods, with predicted bandgaps always below 1 eV. This underestimation is even more severe after performing structure optimization.

GGA functionals generally performed better, predicting higher bandgaps for CBS: ${\left[E_{g}\right]}_{\mathrm{GGA}}^{{\mathrm{LCAO}}_{\mathrm{Exp}}}\in [0.93;1.31]eV$ ${\left[E_{g}\right]}_{\mathrm{GGA}}^{{\mathrm{PAW}}_{\mathrm{Exp}}}\in [0.77;0.99]eV$ with BLYP, and XLYP, aligning best with experimental values. After structural optimization, most GGA functionals predict similar bandgap values, with deviations ≲8% for LCAO-relaxed structures and ≲13% for PAW-relaxed structures.

The hybrid HSE06 functional, known for its accuracy in semiconductor bandgap predictions, drastically overestimated the bandgap with ${\left[E_{g}\right]}_{HSE06}^{{\mathrm{LCAO}}_{\mathrm{Exp}}}=1.9\;eV$, with little changes after structural optimization.

Although the PAW method generally provided slightly more accurate lattice parameters than LCAO, the best overall agreement with experimental values was achieved using the PW91 $\left({\langle ∆\rangle }_{\mathrm{PW}91}^{{\mathrm{LCAO}}_{\mathrm{DFT}}}=1.2\%\right)$ and HSE06 $\left({\langle ∆\rangle }_{\mathrm{HSE}06}^{{\mathrm{LCAO}}_{\mathrm{DFT}}}=0.9\%\right)$ functionals computed with LCAO.

Given its balance between accuracy and computational efficiency (as shown in Figures S11 and S12 and Table S1), we recommend the PW91 functional computed with LCAO for structural optimizations in large supercell studies of dopants and vacancies. For optoelectronic properties, BLYP and XLYP computed with LCAO offer reliable bandgap estimates at reasonable computational costs. This approach, where a cost-efficient functional is used for structural optimization and a more sophisticated one for electronic properties, is a common strategy in DFT, analogous to the frequent pairing of PBE for structural relaxations and HSE06 for bandgap predictions.

In summary, this work establishes a comprehensive framework to select suitable DFT methodologies and XC functionals for CBS, providing key insights to advance both theoretical and experimental studies of this promising PV material. While these findings offer valuable guidance for future CBS research, further exploration of DFT+U corrections, many-body methods (e.g., GW), and alternative hybrid functionals, as well as the addition of spin-orbit effects, remain an open avenue.

## Figures and Tables

**Table 1:** Summary of Cu_3_BiS_3_ key bandgap properties calculated using the LCAO method for different functionals, considering the experimental Wittichenite structure.

LCAO Experimental Wittichenite Structure						
XC Functional		$\mathrm{Indirect}\;E_{g}$	$\mathrm{Direct}\;E_{g}$	$E_{g}$ Type	$E_{F}-CBM$	$VBM-E_{F}$
		(eV)	(eV)		(eV)	(eV)
**LDA**	**HL**	0.93	1.00	Indirect	0.49	−0.44
	**PW**	0.93	0.99	Indirect	0.49	−0.44
	**PZ**	0.93	0.99	Indirect	0.49	−0.44
	**RPA**	0.92	0.98	Indirect	0.48	−0.43
	**Wigner**	0.95	1.01	Indirect	0.50	−0.45
	**XA**	0.81	0.91	Indirect	0.42	−0.39
**GGA**	**BLYP**	1.26	1.35	Indirect	0.66	−0.60
	**BP86**	1.17	1.25	Indirect	0.61	−0.56
	**BPW91**	1.10	1.18	Indirect	0.57	−0.53
	**GAM**	0.93	1.02	Indirect	0.49	−0.45
	**KT2**	1.27	1.36	Indirect	0.63	−0.63
	**PBE**	1.11	1.19	Indirect	0.58	−0.53
	**PBEsol**	1.01	1.08	Indirect	0.53	−0.48
	**PW91**	1.12	1.20	Indirect	0.59	−0.54
	**RPBE**	1.11	1.18	Indirect	0.58	−0.53
	**XLYP**	1.31	1.40	Indirect	0.66	−0.66
**Meta GGA**	**SCAN**	0.65	0.68	Indirect	0.35	−0.30
	**R2SCAN**	0.71	0.75	Indirect	0.38	−0.33
**Hybrid**	**HSE06**	1.92	2.03	Indirect	0.96	−0.96

**Table 2:** Hole and electron effective masses ($m_{h}^{*}$ and $m_{e}^{*}$) calculated using the LCAO method at the VBM and CBM, respectively, considering Cu_3_BiS_3_ experimental Wittichenite structure. When the VBM occurs at the Γ point, the hole masses are calculated for the X, Y and Z directions, designated as $\Gamma_{X}$, $\Gamma_{Y}$ and $\Gamma_{Z}$, respectively. $m_{0}$ is the free electron mass.

LCAO Experimental Wittichenite Structure									
XC Functional		$\frac{m_{h}^{*}}{m_{0}}$	Symmetry Point	$\frac{m_{h}^{*}}{m_{0}}$	Symmetry Point	$\frac{m_{h}^{*}}{m_{0}}$	Symmetry Point	$\frac{m_{e}^{*}}{m_{0}}$	Symmetry Point
**LDA**	**HL**	−1.3	$\Gamma_{X}$	−0.4	$\Gamma_{Y}$	−1.4	$\Gamma_{Z}$	0.6	T
	**PW**	−1.4		−0.5		−1.4		0.6	
	**PZ**	−1.3		−0.5		−1.4		0.6	
	**RPA**	−1.3		−0.5		−1.4		0.6	
	**Wigner**	−1.3		−0.4		−1.4		0.6	
	**XA**	−1.6		−0.8		−1.9		0.6	
**GGA**	**BLYP**	−1.4	$\Gamma_{X}$	−0.4	$\Gamma_{Y}$	−1.5	$\Gamma_{Z}$	0.6	T
	**BP86**	−1.4		−0.4		−1.5		0.6	
	**BPW91**	−1.4		−0.5		−1.4		0.6	
	**GAM**	−1.5		−0.5		−1.5		0.6	
	**KT2**	−1.3		−0.5		−1.5		0.6	
	**PBE**	−1.3		−0.4		−1.5		0.6	
	**PBEsol**	−1.3		−0.5		−1.4		0.6	
	**PW91**	−1.3		−0.5		−1.5		0.6	
	**RPBE**	−1.4		−0.4		−1.4		0.6	
	**XLYP**	−1.4		−0.4		−1.5		0.6	
**Meta** **GGA**	**SCAN**	−1.3	$\Gamma_{X}$	−0.5	$\Gamma_{Y}$	−1.4	$\Gamma_{Z}$	0.6	T
	**R2SCAN**	−1.4		−0.5		−1.4		0.6	
**Hybrid**	**HSE06**	−1.3	$\Gamma_{X}$	−0.4	$\Gamma_{Y}$	−1.3	$\Gamma_{Z}$	0.5	T

**Table 3:** Summary of Cu_3_BiS_3_ key bandgap properties calculated using the PAW method for different functionals, considering Cu_3_BiS_3_ experimental Wittichenite structure.

PAW Experimental Wittichenite Structure						
XC Functional		$\mathrm{Indirect}\;E_{g}$	$\mathrm{Direct}\;E_{g}$	$E_{g}$ Type	$E_{F}-CBM$	$VBM-E_{F}$
		(eV)	(eV)		(eV)	(eV)
**LDA**	**HL**	0.77	0.83	Indirect	0.41	−0.36
	**PW**	0.78	0.83	Indirect	0.42	−0.36
	**PZ**	0.78	0.83	Indirect	0.42	−0.36
	**RPA**	0.78	0.83	Indirect	0.42	−0.36
	**Wigner**	0.73	0.77	Indirect	0.39	−0.34
	**XA**	1.62	1.77	Indirect	0.84	−0.78
**GGA**	**BLYP**	0.86	0.93	Indirect	0.45	−0.40
	**BP86**	0.83	0.89	Indirect	0.44	−0.39
	**BPW91**	0.82	0.88	Indirect	0.44	−0.38
	**GAM**	0.99	1.09	Indirect	0.52	−0.47
	**KT2**	0.79	0.85	Indirect	0.42	−0.37
	**PBE**	0.81	0.87	Indirect	0.43	−0.38
	**PBEsol**	0.77	0.83	Indirect	0.41	−0.36
	**PW91**	0.81	0.87	Indirect	0.43	−0.38
	**RPBE**	0.84	0.90	Indirect	0.44	−0.39
	**XLYP**	0.87	0.94	Indirect	0.46	−0.41

**Table 4:** Hole and electron effective masses ($m_{h}^{*}$ and $m_{e}^{*}$) calculated using the PAW method at the VBM and CBM, respectively, considering Cu_3_BiS_3_ experimental Wittichenite structure. When the VBM occurs at the Γ point, the hole masses are calculated for the X, Y and Z directions, designated as $\Gamma_{X}$, $\Gamma_{Y}$ and $\Gamma_{Z}$, respectively. $m_{0}$ is the free electron mass.

PAW Experimental Wittichenite Structure									
XC Functional		$\frac{m_{h}^{*}}{m_{0}}$	Symmetry Point	$\frac{m_{h}^{*}}{m_{0}}$	Symmetry Point	$\frac{m_{h}^{*}}{m_{0}}$	Symmetry Point	$\frac{m_{e}^{*}}{m_{0}}$	Symmetry Point
**LDA**	**HL**	−1.3	$\Gamma_{X}$	−0.4	$\Gamma_{Y}$	−1.4	$\Gamma_{Z}$	0.6	T
	**PW**	−1.3		−0.4		−1.4		0.6	
	**PZ**	−1.3		−0.4		−1.5		0.6	
	**RPA**	−1.3		−0.4		−1.5		0.6	
	**Wigner**	−1.2		−0.4		−1.3		0.6	
	**XA**	−1.6		−0.6		−2.0		0.6	
**GGA**	**BLYP**	−1.4	$\Gamma_{X}$	−0.4	$\Gamma_{Y}$	−1.5	$\Gamma_{Z}$	0.6	T
	**BP86**	−1.4		−0.4		−1.5		0.6	
	**BPW91**	−1.4		−0.4		−1.5		0.6	
	**GAM**	−1.3		−0.5		−1.6		0.6	
	**KT2**	−1.3		−0.4		−1.5		0.5	
	**PBE**	−1.4		−0.4		−1.4		0.6	
	**PBEsol**	−1.3		−0.4		−1.4		0.5	
	**PW91**	−1.3		−0.4		−1.4		0.6	
	**RPBE**	−1.4		−0.4		−1.4		0.6	
	**XLYP**	−1.4		−0.4		−1.5		0.6	

**Table 5:** Comparison of lattice parameters for different CBS P2_1_2_1_2_1_ Wittichenite structures. The bold PDF# corresponds to the experimental CBS structure used as a reference in this study. ∆a, ∆b, and ∆c denote the relative deviations of each lattice parameter from the reference structure. All measurements were taken at near room temperature.

Experimental Lattice Parameters								
PDF #	a (Å)	b (Å)	c (Å)	∆a	∆b	∆c	T (K)	Reference
**04-006-8325**	**7.723**	**10.395**	**6.716**	**Used in this work**			**298**	**Kocman et al. [95]**
00-043-1479	7.696	10.388	6.712	−0.3%	−0.1%	−0.1%	298	Kocman et al. [95]
01-073-1185	7.700	10.410	6.740	−0.3%	0.1%	0.4%	298	Matzat et al. [96]
01-087-7691	7.657	10.308	6.707	−0.9%	−0.8%	−0.1%	298	Criddle et al. [97]
04-004-0452	7.696	10.364	6.729	−0.3%	−0.3%	0.2%	300	Makovicky et al. [98]

**Table 6:** Summary of the lattice parameters of the CBS P2_1_2_1_2_1_ Wittichenite structures predicted using DFT calculations with the LCAO method for several XC functionals. ∆a, ∆b and ∆c represent the relative difference between the predicted structures and the reference experimental Wittichenite structure. $\langle ∆\rangle$ denotes the average absolute relative deviation across all lattice parameters. Functionals marked with “*” did not achieve the 0.01 eV/Å convergence tolerance.

LCAO Relaxed Structure Lattice Parameters								
XC Functional		a(Å)	b (Å)	c(Å)	∆a	∆b	∆c	$\langle ∆\rangle =\frac{\left|∆a\right|+\left|∆b\right|+\left|∆c\right|}{3}$
**LDA**	**HL**	7.3503	10.4735	6.1834	−4.8%	0.8%	−7.9%	4.5%
	**PW**	7.3526	10.4649	6.1793	−4.8%	0.7%	−8.0%	4.5%
	**PZ**	7.3507	10.4658	6.1794	−4.8%	0.7%	−8.0%	4.5%
	**RPA**	7.2791	10.4398	6.0903	−5.7%	0.4%	−9.3%	5.2%
	**Wigner**	7.3987	10.5766	6.2677	−4.2%	1.7%	−6.7%	4.2%
	**XA**	6.2203	9.4477	5.7470	−19.5%	−9.1%	−14.4%	14.3%
**GGA**	**BLYP**	8.3722	10.5876	7.0077	8.4%	1.9%	4.3%	4.9%
	**BP86**	7.8257	10.6345	6.8938	1.3%	2.3%	2.6%	2.1%
	BPW91 *	7.5757	10.6488	6.8786	−1.9%	2.4%	2.4%	2.3%
	GAM *	8.1452	10.7713	7.0062	5.5%	3.6%	4.3%	4.5%
	**KT2**	7.2425	10.5516	6.2008	−6.2%	1.5%	−7.7%	5.1%
	**PBE**	7.9564	10.7154	7.0433	3.0%	3.1%	4.9%	3.7%
	**PBEsol**	7.3934	10.5412	6.3001	−4.3%	1.4%	−6.2%	4.0%
	**PW91**	7.6544	10.5126	6.8310	−0.9%	1.1%	1.7%	1.2%
	**RPBE**	7.9153	10.5593	6.9430	2.5%	1.6%	3.4%	2.5%
	**XLYP**	8.1451	10.7715	7.1114	5.5%	3.6%	5.9%	5.0%
**Meta** **GGA**	**SCAN**	7.1469	10.5251	6.7512	−7.5%	1.3%	0.5%	3.1%
	**R2SCAN**	7.4277	10.5544	6.7057	−3.8%	1.5%	−0.2%	1.8%
**Hybrid**	**HSE06**	7.7010	10.4879	6.8131	−0.3%	0.9%	1.4%	0.9%

**Table 7:** Summary of Cu_3_BiS_3_ key bandgap properties calculated using the LCAO method for different functionals, considering their relaxed structures. Functionals marked with “*” did not achieve the 0.01 eV/Å convergence tolerance.

LCAO Relaxed Structure						
XC Functional		$\mathrm{Indirect}\;E_{g}$	$\mathrm{Direct}\;E_{g}$	$E_{g}$ Type	$E_{F}-CBM$	$VBM-E_{F}$
		(eV)	(eV)		(eV)	(eV)
**LDA**	**HL**	0.49	0.95	Indirect	0.26	−0.23
	**PW**	0.49	0.95	Indirect	0.26	−0.23
	**PZ**	0.49	0.95	Indirect	0.26	−0.23
	**RPA**	0.43	0.90	Indirect	0.23	−0.19
	**Wigner**	0.52	0.95	Indirect	0.27	−0.25
	**XA**	0.29	0.78	Indirect	0.17	−0.12
**GGA**	**BLYP**	1.23	1.32	Indirect	0.62	−0.62
	**BP86**	1.11	1.20	Indirect	0.57	−0.54
	BPW91 *	1.06	1.17	Indirect	0.54	−0.52
	GAM *	0.89	0.97	Indirect	0.46	−0.43
	**KT2**	0.77	1.29	Indirect	0.41	−0.36
	**PBE**	1.11	1.14	Indirect	0.57	−0.53
	**PBEsol**	0.60	1.00	Indirect	0.31	−0.30
	**PW91**	1.03	1.09	Indirect	0.54	−0.49
	**RPBE**	1.01	1.08	Indirect	0.52	−0.49
	**XLYP**	1.32	1.38	Indirect	0.66	−0.66
**Meta GGA**	**SCAN**	0.52	0.60	Indirect	0.28	−0.25
	**R2SCAN**	0.60	0.70	Indirect	0.32	−0.28
**Hybrid**	**HSE06**	2.01	2.14	Indirect	1.01	−1.01

**Table 8:** Hole and electron effective masses ($m_{h}^{*}$ and $m_{e}^{*}$) calculated using the LCAO method at the VBM and CBM, respectively, for the different functionals considering their relaxed structures. When the VBM occurs at the Γ point, the hole masses are calculated for the X, Y and Z directions, designated as $\Gamma_{X}$, $\Gamma_{Y}$ and $\Gamma_{Z}$, respectively. $m_{0}$ is the free electron mass. Functionals marked with “*” did not achieve the 0.01 eV/Å convergence tolerance. N.A. stands for not applicable.

LCAO Relaxed Structure									
XC Functional		$\frac{m_{h}^{*}}{m_{0}}$	Symmetry Point	$\frac{m_{h}^{*}}{m_{0}}$	Symmetry Point	$\frac{m_{h}^{*}}{m_{0}}$	Symmetry Point	$\frac{m_{e}^{*}}{m_{0}}$	Symmetry Point
**LDA**	**HL**	−2.0	$\Gamma_{X}$	−0.5	$\Gamma_{Y}$	−0.7	$\Gamma_{Z}$	0.6	T
	**PW**	−2.0		−0.5		−0.7		0.6	
	**PZ**	−2.0		−0.5		−0.7		0.6	
	**RPA**	−1.5		−0.5		−0.7		0.4	Z
	**Wigner**	−2.5		−0.5		−0.8		0.6	T
	**XA**	−15.9		−1.0		−0.9		0.4	910ΓZ¯
**GGA**	**BLYP**	−1.6	$\Gamma_{X}$	−0.4	$\Gamma_{Y}$	−2.0	$\Gamma_{Z}$	0.9	35ΓZ¯
	**BP86**	−1.5		−0.5		−1.6		0.6	T
	BPW91 *	−22.9		−0.6		−1.5		0.7	
	GAM *	−2.4		−0.5		−2.0		0.6	
	**KT2**	−1.0		−0.5		−0.7		0.4	910ΓZ¯
	**PBE**	−1.5		−0.5		−1.6		0.7	T
	**PBEsol**	−3.1		−0.6		−0.9		0.6	
	**PW91**	−1.6		−0.5		−1.5		0.7	
	**RPBE**	−1.4		−0.5		−1.6		0.6	
	**XLYP**	−1.4		−0.5		−1.8		0.8	23ΓZ¯
**Meta** **GGA**	**SCAN**	−0.5	23ΓX¯	N.A.		N.A.		0.7	910ΓZ¯
	**R2SCAN**	−1.0	35ΓX¯					0.8	T
**Hybrid**	**HSE06**	−1.9	$\Gamma_{X}$	−0.5	$\Gamma_{Y}$	−1.4	$\Gamma_{Z}$	0.5	T

**Table 9:** Summary of the lattice parameters of the CBS P2_1_2_1_2_1_ Wittichenite structures predicted using DFT calculations with the PAW method for several XC functionals. ∆a, ∆b**,** and ∆c represent the relative difference between the predicted structures and the reference experimental Wittichenite structure. $\langle ∆\rangle$ denotes the average absolute relative deviation across all lattice parameters.

PAW Relaxed Structure Lattice Parameters								
XC Functional		a(Å)	b (Å)	c(Å)	∆a	∆b	∆c	$\langle ∆\rangle =\frac{\left|∆a\right|+\left|∆b\right|+\left|∆c\right|}{3}$
**LDA**	**HL**	7.3608	10.4337	6.2136	−4.7%	0.4%	−7.5%	4.2%
	**PW**	7.3591	10.4233	6.2148	−4.7%	0.3%	−7.5%	4.1%
	**PZ**	7.3591	10.4268	6.2111	−4.7%	0.3%	−7.5%	4.2%
	**RPA**	7.2942	10.3717	6.1192	−5.6%	−0.2%	−8.9%	4.9%
	**Wigner**	7.4516	10.5317	6.3072	−3.5%	1.3%	−6.1%	3.6%
	**XA**	6.2844	9.4626	5.7058	−18.6%	−9.0%	−15.0%	14.2%
**GGA**	**BLYP**	8.2990	10.4876	7.0063	7.5%	0.9%	4.3%	4.2%
	**BP86**	7.8733	10.5216	6.8619	1.9%	1.2%	2.2%	1.8%
	**BPW91**	7.6002	10.5215	6.7696	−1.6%	1.2%	0.8%	1.2%
	**GAM**	8.2758	10.4038	6.9232	7.2%	0.1%	3.1%	3.4%
	**KT2**	7.3602	10.3814	6.1632	−4.7%	−0.1%	−8.2%	4.4%
	**PBE**	7.5335	10.5490	6.7281	−2.5%	1.5%	0.2%	1.4%
	**PBEsol**	7.4014	10.4420	6.2253	−4.2%	0.5%	−7.3%	4.0%
	**PW91**	7.5436	10.5282	6.7515	−2.3%	1.3%	0.5%	1.4%
	**RPBE**	7.8264	10.4997	6.8274	1.3%	1.0%	1.7%	1.3%
	**XLYP**	8.4712	10.5177	7.0292	9.7%	1.2%	4.7%	5.2%

**Table 10:** Summary of Cu_3_BiS_3_ key bandgap properties calculated using the PAW method for different functionals, considering their relaxed structures.

PAW Relaxed Structure						
XC Functional		$\mathrm{Indirect}\;E_{g}$	$\mathrm{Direct}\;E_{g}$	$E_{g}$ Type	$E_{F}-CBM$	$VBM-E_{F}$
		(eV)	(eV)		(eV)	(eV)
**LDA**	**HL**	0.39	0.81	Indirect	0.21	−0.18
	**PW**	0.40	0.82	Indirect	0.21	−0.19
	**PZ**	0.40	0.82	Indirect	0.21	−0.19
	**RPA**	0.35	0.83	Indirect	0.20	−0.15
	**Wigner**	0.37	0.74	Indirect	0.19	−0.18
	**XA**	1.02	1.49	Indirect	0.51	−0.51
**GGA**	**BLYP**	0.83	0.86	Indirect	0.43	−0.40
	**BP86**	0.75	0.78	Indirect	0.40	−0.35
	**BPW91**	0.71	0.83	Indirect	0.37	−0.34
	**GAM**	1.15	1.23	Indirect	0.59	−0.56
	**KT2**	0.39	0.86	Indirect	0.21	−0.18
	**PBE**	0.71	0.86	Indirect	0.36	−0.34
	**PBEsol**	0.35	0.77	Indirect	0.19	−0.16
	**PW91**	0.71	0.84	Indirect	0.37	−0.34
	**RPBE**	0.72	0.78	Indirect	0.38	−0.34
	**XLYP**	0.85	0.91	Indirect	0.44	−0.41

**Table 11:** Hole and electron effective masses ($m_{h}^{*}$ and $m_{e}^{*}$) calculated using the PAW method at the VBM and CBM, respectively, for the different functionals considering their relaxed structures. When the VBM occurs at the Γ point, the hole masses are calculated for the X, Y and Z directions, designated as $\Gamma_{X}$, $\Gamma_{Y}$ and $\Gamma_{Z}$, respectively. $m_{0}$ is the free electron mass.

PAW Relaxed Structure									
XC Functional		$\frac{m_{h}^{*}}{m_{0}}$	Symmetry Point	$\frac{m_{h}^{*}}{m_{0}}$	Symmetry Point	$\frac{m_{h}^{*}}{m_{0}}$	Symmetry Point	$\frac{m_{e}^{*}}{m_{0}}$	Symmetry Point
**LDA**	**HL**	−2.9	$\Gamma_{X}$	−0.5	$\Gamma_{Y}$	−0.7	$\Gamma_{Z}$	0.5	T
	**PW**	−3.1		−0.5		−0.8		0.5	
	**PZ**	−3.7		−0.5		−0.7		0.6	
	**RPA**	−1.8		−0.5		−0.6		0.6	
	**Wigner**	−8.3		−0.6		−0.8		0.5	
	**XA**	−1.2		−1.3		−1.4		0.3	910ΓZ¯
**GGA**	**BLYP**	−1.3	$\Gamma_{X}$	−0.4	$\Gamma_{Y}$	−1.9	$\Gamma_{Z}$	1.0	35ΓZ¯
	**BP86**	−1.9		−0.4		−1.5		0.6	T
	**BPW91**	−19.9		−0.5		−1.4		0.6	
	**GAM**	−1.5		−0.5		−1.9		1.1	12ΓZ¯
	**KT2**	−2.3		−0.6		−0.7		0.6	T
	**PBE**	−1.8	25ΓX¯	N.A.		N.A.		0.6	
	**PBEsol**	−3.6	$\Gamma_{X}$	−0.5	$\Gamma_{Y}$	−0.7	$\Gamma_{Z}$	0.5	
	**PW91**	−2.5	13ΓX¯	N.A.		N.A.		0.6	
	**RPBE**	−2.1	$\Gamma_{X}$	−0.4	$\Gamma_{Y}$	−1.5	$\Gamma_{Z}$	0.6	
	**XLYP**	−1.4		−0.4		−2.0		1.0	12ΓZ¯

## Data Availability

The original contributions presented in this study are included in the article/Supplementary Materials. Further inquiries can be directed to the corresponding author.

## Supplementary Materials

The following supporting information can be downloaded at: https://www.mdpi.com/article/10.3390/ma18061213/s1, Figure S1—Stacked Partial Density of States calculated using the LCAO method for different LDA functionals considering Cu3BiS3 experimental Wittichenite structure. Figure S2—Stacked Partial Density of States calculated using the LCAO method for different GGA functionals considering Cu3BiS3 experimental Wittichenite structure. Figure S3—Stacked Partial Density of States calculated using the LCAO method for different meta-GGA and Hybrid functionals considering Cu3BiS3 experimental Wittichenite structure. Figure S4—Stacked Partial Density of States calculated using the PAW method for different LDA functionals considering Cu3BiS3 experimental Wittichenite structure. Figure S5—Stacked Partial Density of States calculated using the PAW method for different GGA functionals considering Cu3BiS3 experimental Wittichenite structure. Figure S6—Stacked Partial Density of States calculated using the LCAO method for different LDA functionals considering relaxed structures. Figure S7—Stacked Partial Density of States calculated using the LCAO method for different GGA functionals considering relaxed structures. Figure S8—Stacked Partial Density of States calculated using the LCAO method for different meta-GGA and Hybrid functionals considering relaxed structures. Figure S9—Stacked Partial Density of States calculated using the PAW method for different LDA functionals considering relaxed structures. Figure S10—Stacked Partial Density of States calculated using the PAW method for different GGA functionals considering relaxed structures. Table S1—Computation time of the different DFT methods (LCAO and PAW) for all the considered XC functionals. The relative computational cost between the PAW and LCAO methods is also presented. Figure S11—Computation time of the different DFT methods (LCAO and PAW) for all the considered XC functionals. Figure S12—Relative computational cost between the PAW and LCAO methods, “PAW”/“LCAO”.
