# Supplementary material for: Assessing Cu3BiS3 for Thin-Film Photovoltaics: A Systematic DFT Study Comparing LCAO and PAW Across Multiple Functionals
Source: Materials (Basel). 2025 Mar 8;18(6):1213. doi: 10.3390/ma18061213 (PMC11943643; doi:10.3390/ma18061213)
Supplement: Supplementary file 1 [file materials-18-01213-s001.zip › materials-3498505-supplementary.pdf]

Supplementary Information

Assessing Cu_3BiS_3 for Thin-Film Photovoltaics: A Systematic DFT Study Comparing LCAO and PAW Across Multiple Functionals

C.O. Amorim*, S.M. Sivasankar, and A.F. da Cunha

Physics Department and i3N, University of Aveiro, Campus de Santiago, 3810-193 Aveiro, Portugal

***Email:** amorim5@ua.pt

Partial Density of States

LCAO Calculations with Experimental Structure

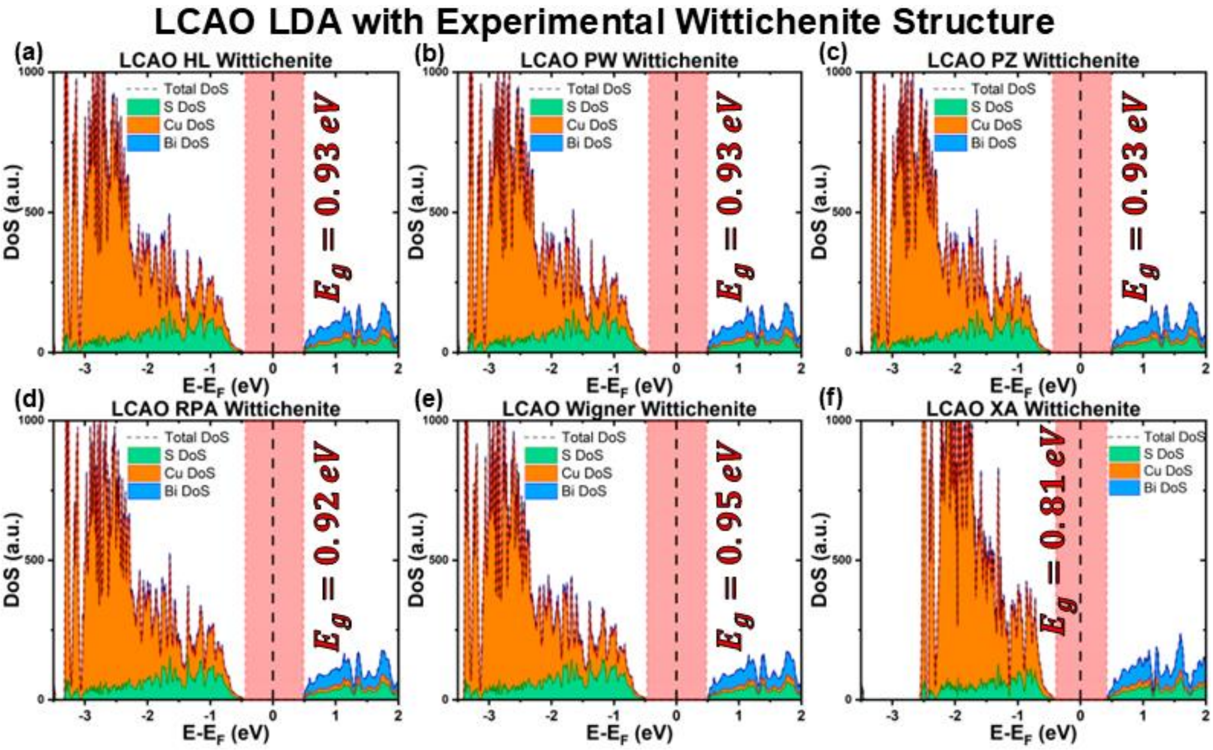

Figure S1 – Stacked Partial Density of States calculated using the LCAO method for different LDA functionals considering Cu_3BiS_3 experimental Wittichenite structure.

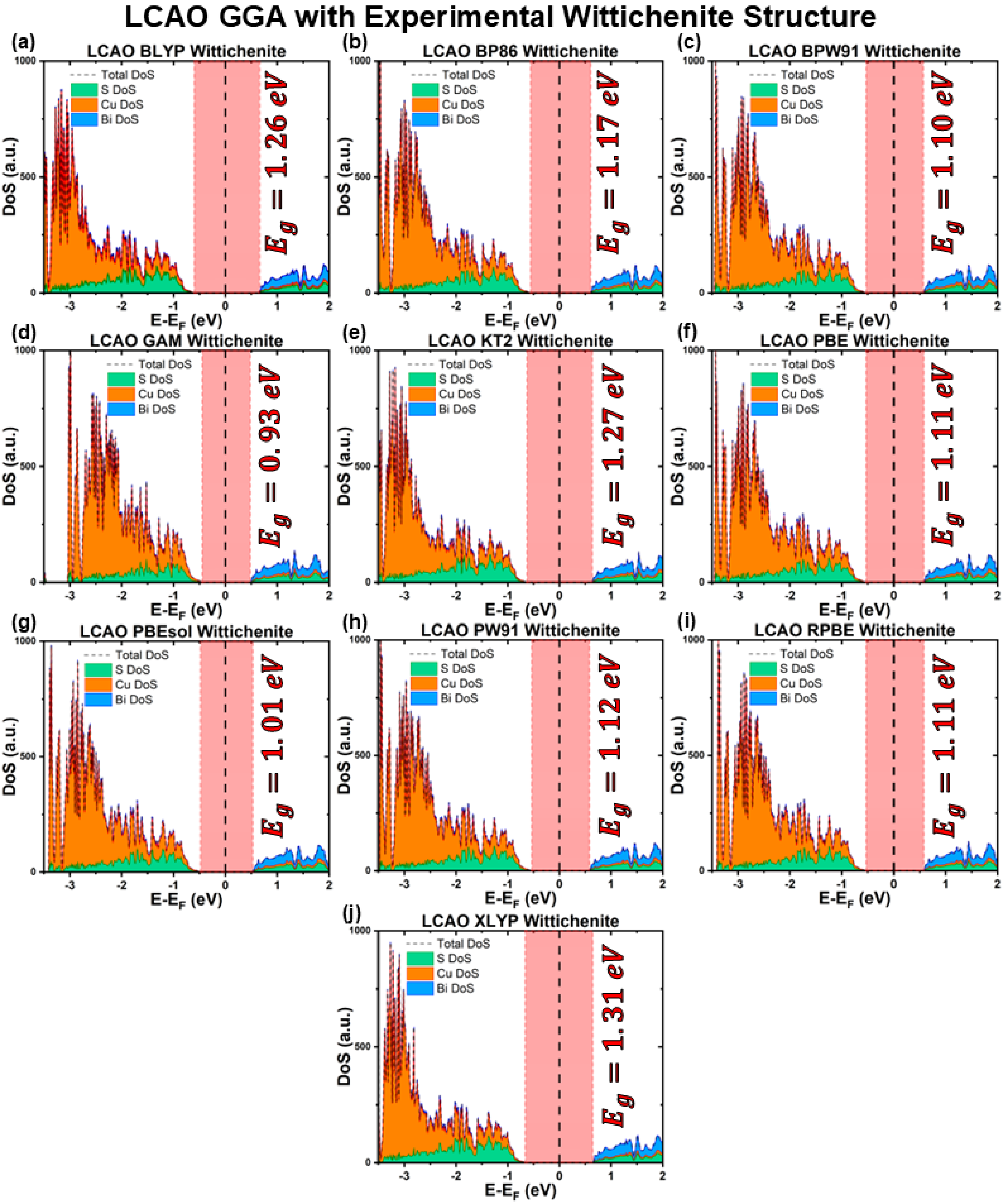

Figure S2 – Stacked Partial Density of States calculated using the LCAO method for different GGA functionals considering Cu_3BiS_3 experimental Wittichenite structure.

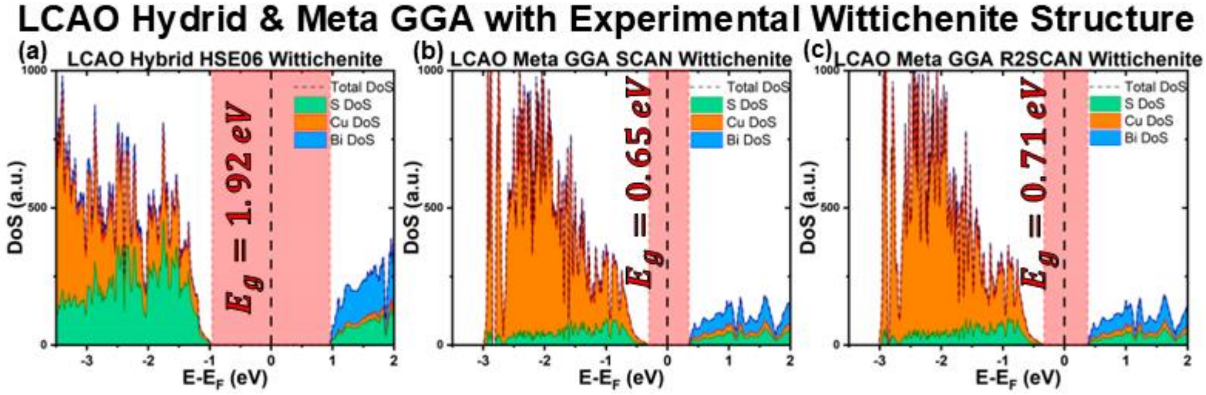

Figure S3 – Stacked Partial Density of States calculated using the LCAO method for different meta-GGA and Hybrid functionals considering Cu_3BiS_3 experimental Wittichenite structure.

PAW Calculations with Experimental Structure

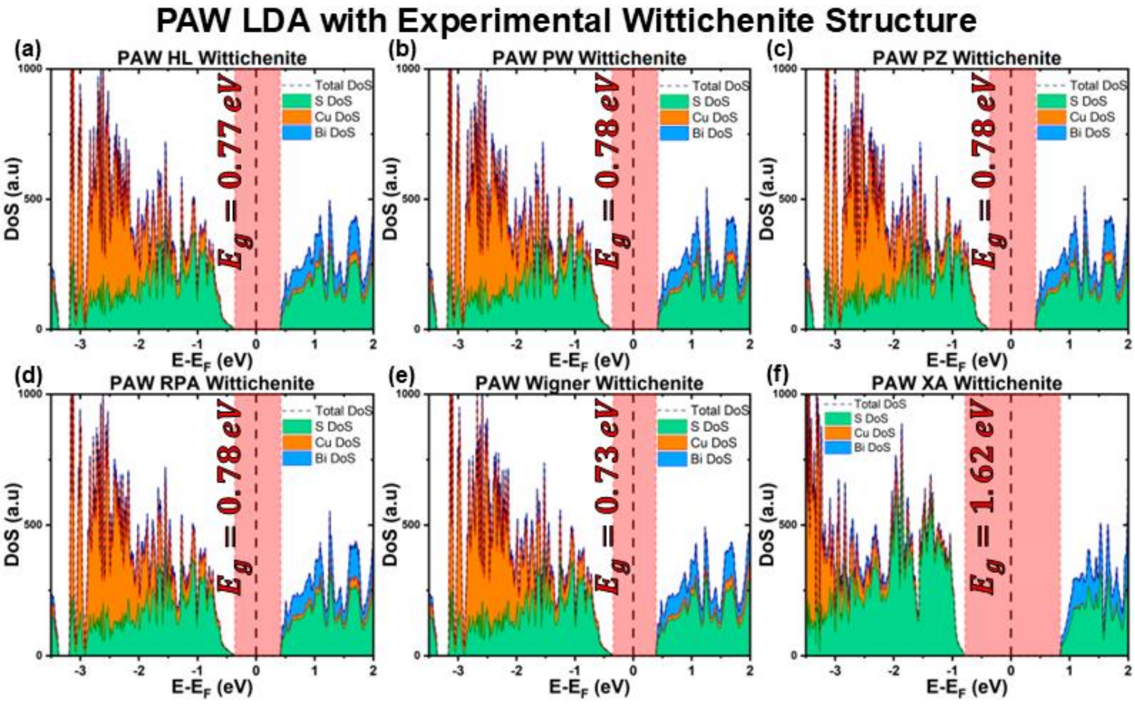

Figure S4 – Stacked Partial Density of States calculated using the PAW method for different LDA functionals considering Cu_3BiS_3 experimental Wittichenite structure.

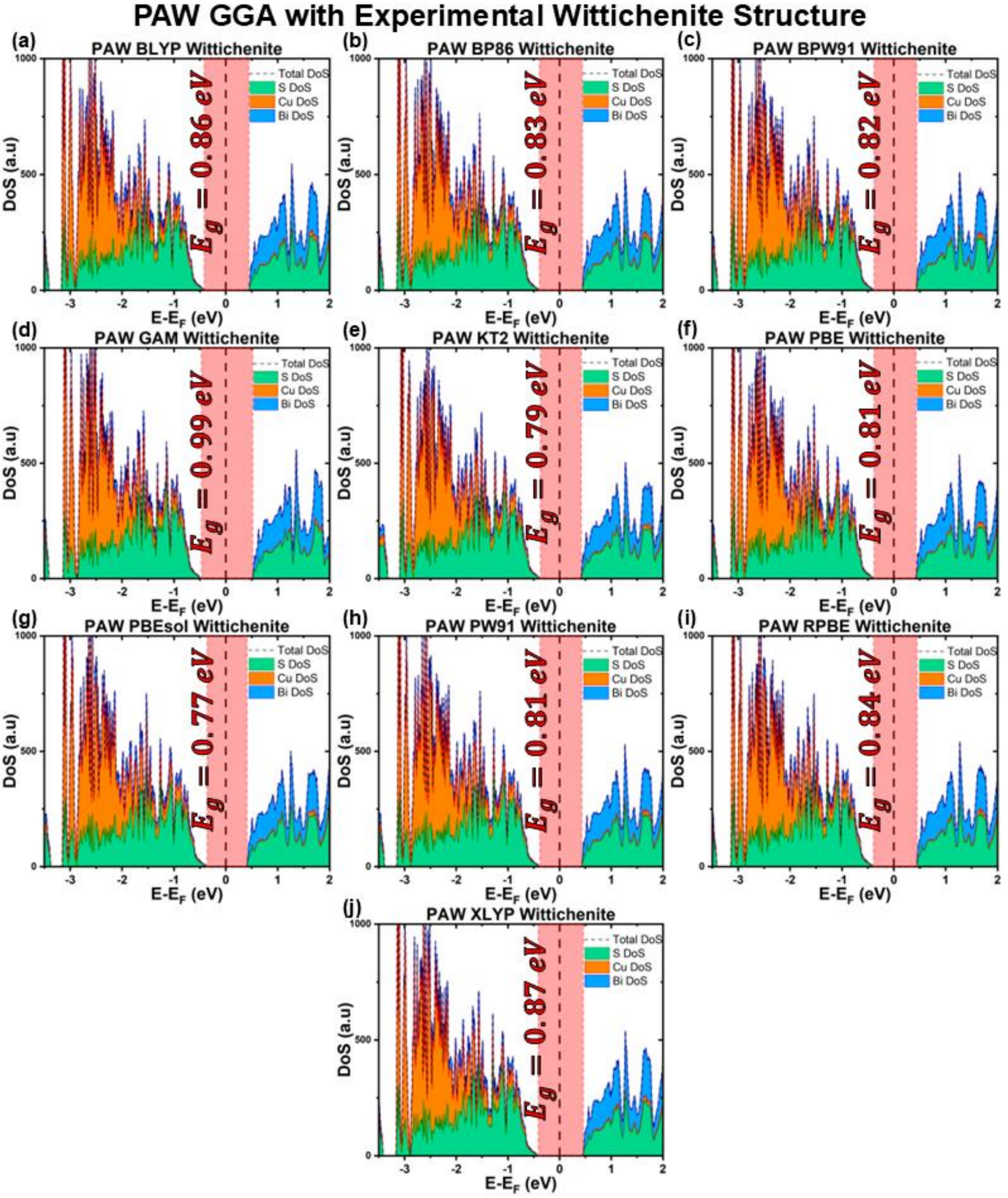

Figure S5 – Stacked Partial Density of States calculated using the PAW method for different GGA functionals considering Cu_3BiS_3 experimental Wittichenite structure.

LCAO Calculations with Fully Relaxed Structure

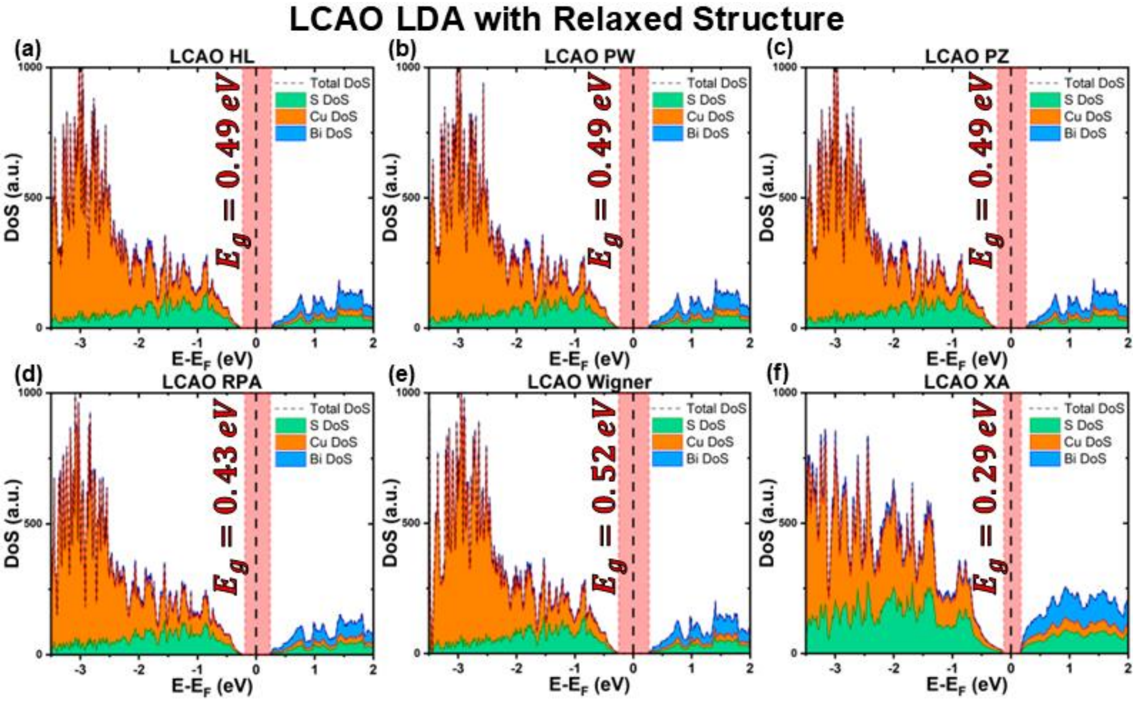

Figure S6 – Stacked Partial Density of States calculated using the LCAO method for different LDA functionals considering relaxed structures.

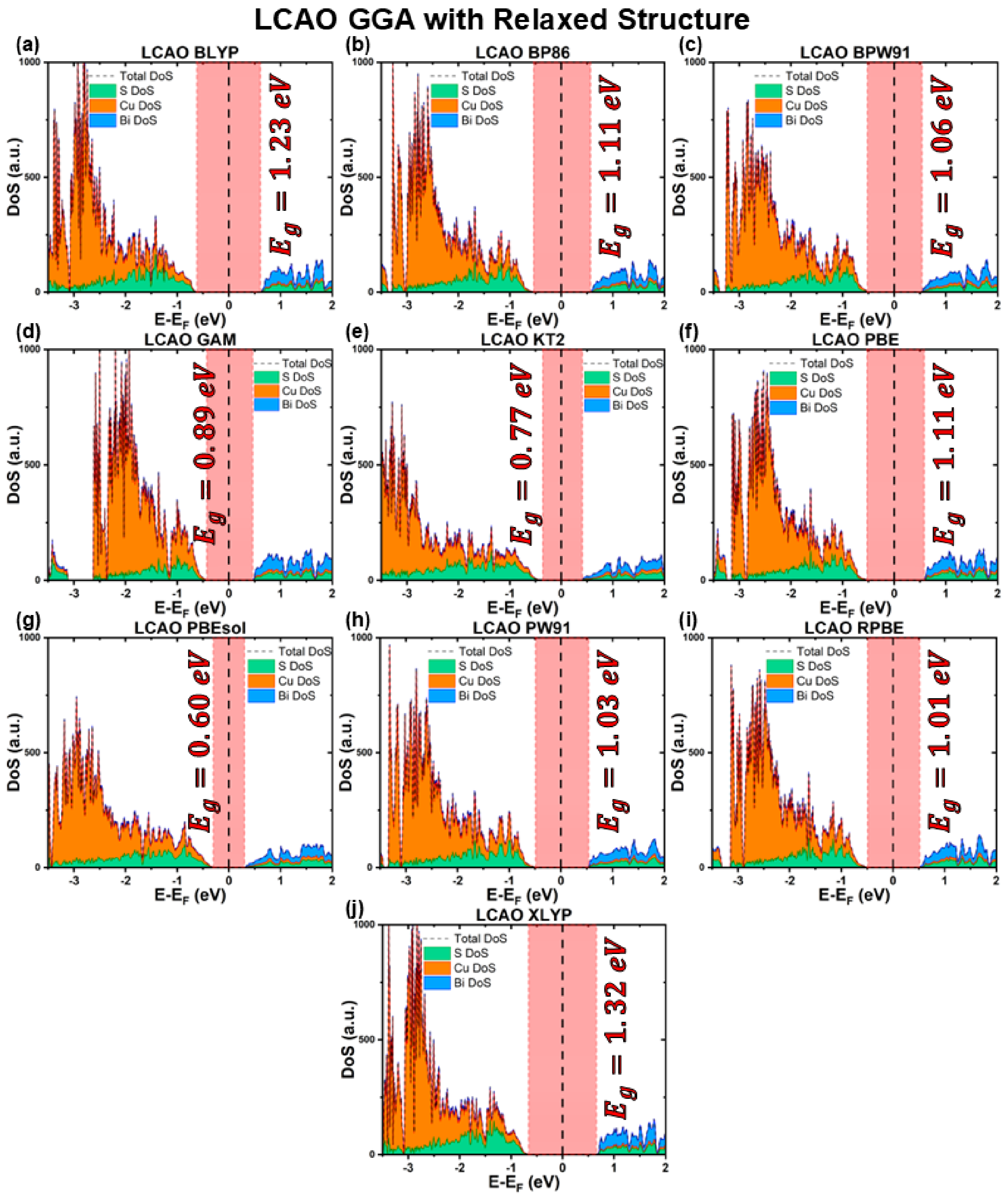

Figure S7 – Stacked Partial Density of States calculated using the LCAO method for different GGA functionals considering relaxed structures.

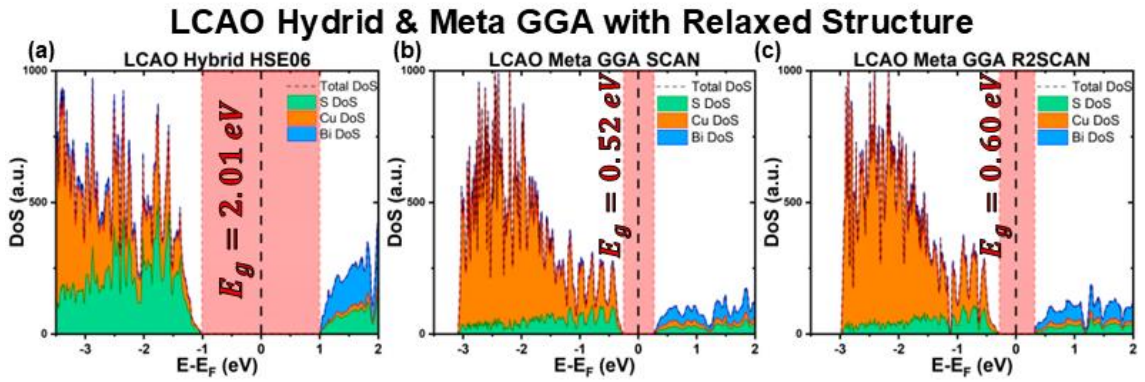

Figure S8 – Stacked Partial Density of States calculated using the LCAO method for different meta-GGA and Hybrid functionals considering relaxed structures.

PAW Calculations with Fully Relaxed Structure

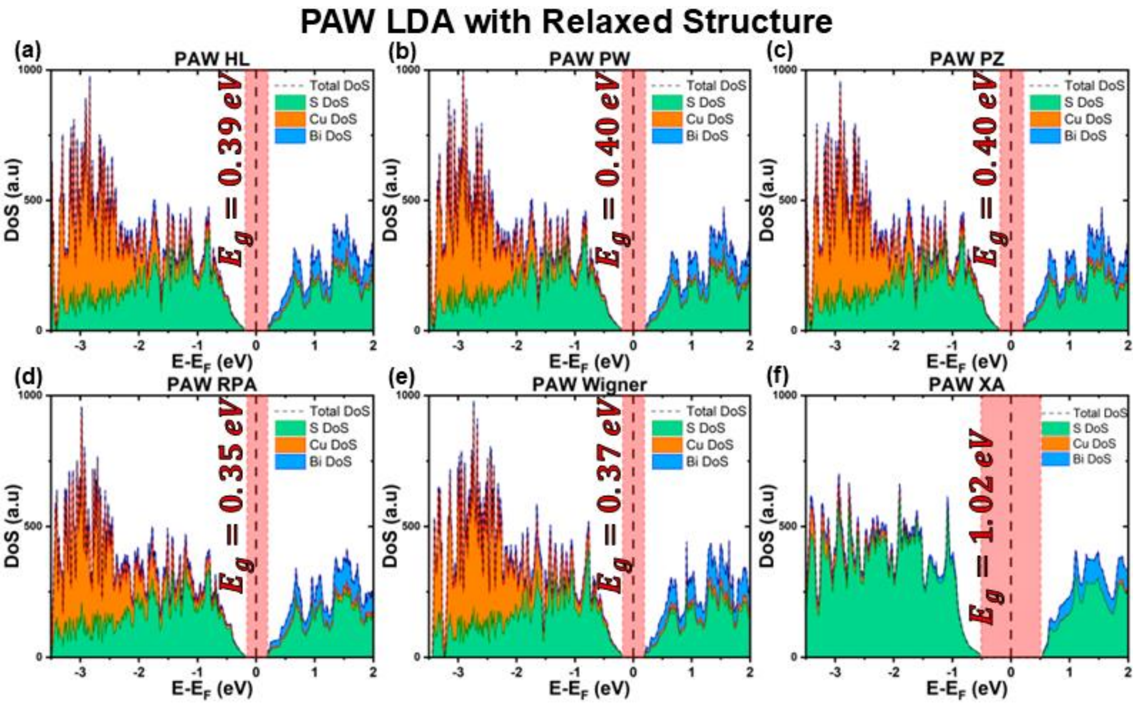

Figure S9 – Stacked Partial Density of States calculated using the PAW method for different LDA functionals considering relaxed structures.

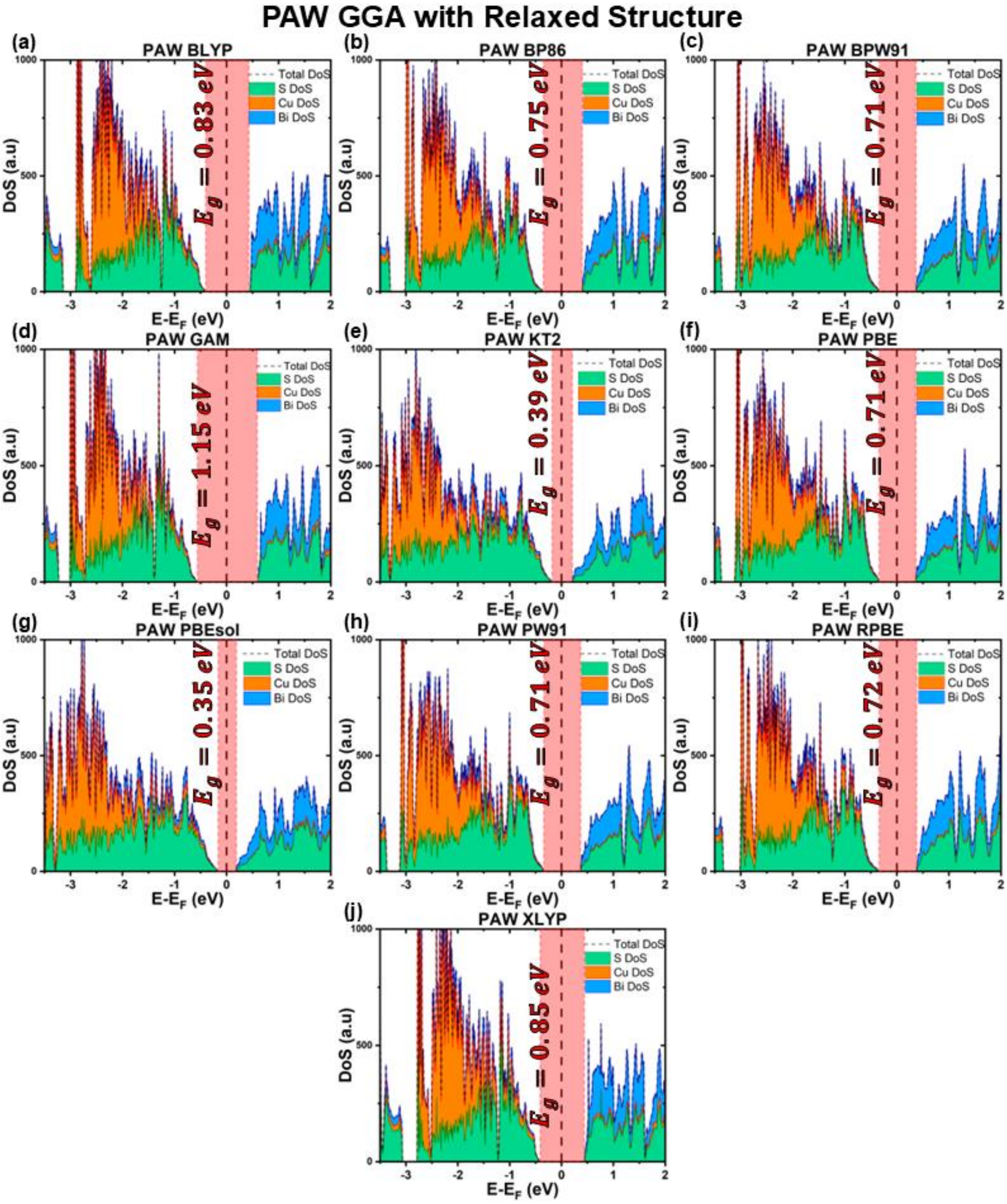

Figure S10 – Stacked Partial Density of States calculated using the PAW method for different GGA functionals considering relaxed structures.

Computational Cost Analysis

To assess the computational efficiency of the LCAO method relative to PAW, we measured the computation time for a single SCF calculation across all considered XC functionals, following the conditions outlined in the Computational Methods section. The results are presented in Table S1 and Figures S11–S12.

Table S1 – Computation time of the different DFT methods (LCAO and PAW) for all the considered XC functionals. The relative computational cost between the PAW and LCAO methods is also presented.

XC Functional		Computation Time (s)		$\frac{\text{PAW}}{\text{LCAO}}$
		LCAO	PAW	
LDA	HL	772.58	16115.26	21
	PW	687.04	12265.51	18
	PZ	693.16	11509.97	17
	RPA	688.92	10289.46	15
	Wigner	678.52	9376.149	14
	XA	751.34	10480.63	14
GGA	BLYP	674.49	36633.44	54
	BP86	707.31	33671.39	48
	BPW91	664.38	40749.17	61
	GAM	875.76	37132.58	42
	KT2	812.00	37641.01	46
	PBE	761.77	40259.58	53
	PBEsol	946.21	39036.70	41
	PW91	720.64	40749.17	57
	RPBE	682.60	40195.45	59
	XLYP	625.73	39661.65	63
Meta GGA	SCAN	1647.08		
	R2SCAN	1698.94		
Hybrid	HSE06	26661.89		

As shown in Table S1 and Figure S11, the LCAO method consistently requires significantly less computational time than the PAW method for the same XC functionals. Additionally, while computation times for LDA and GGA functionals remain comparable within the LCAO framework,

PAW calculations exhibit a substantial increase in computational cost for GGA functionals compared to LDA.

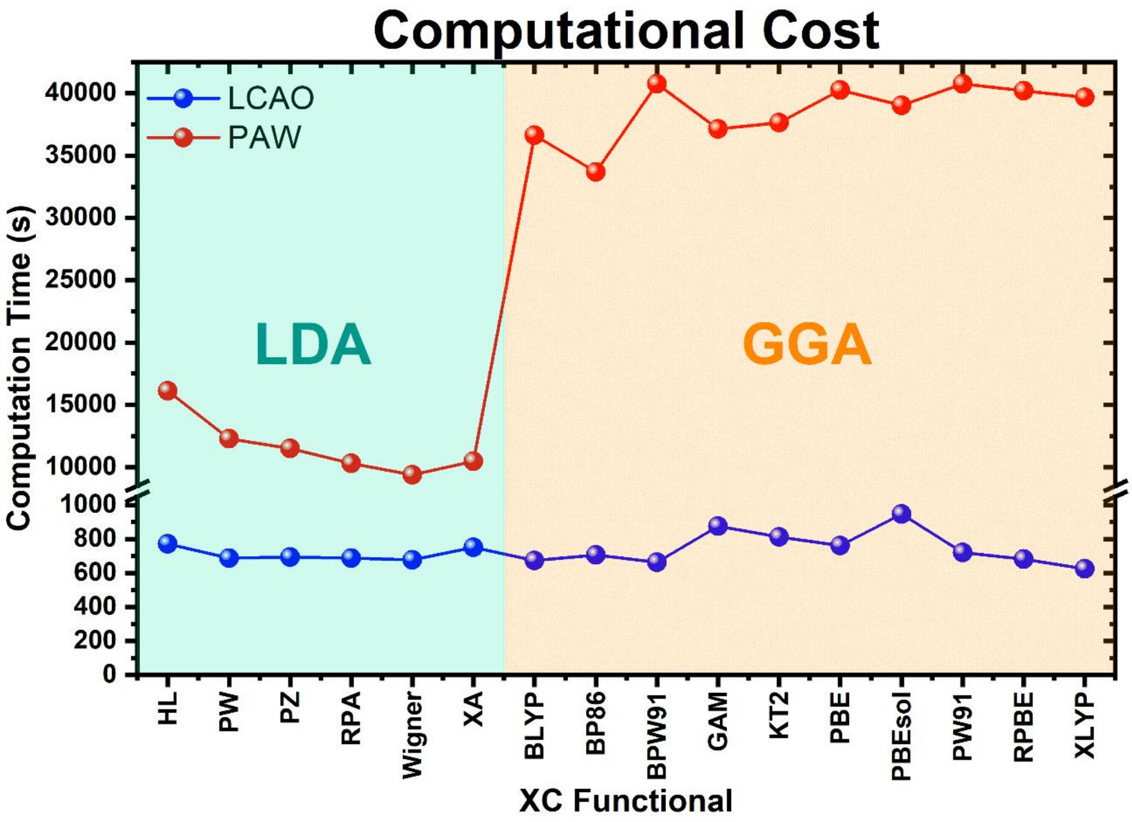

Figure S11 – Computation time of the different DFT methods (LCAO and PAW) for all the considered XC functionals.

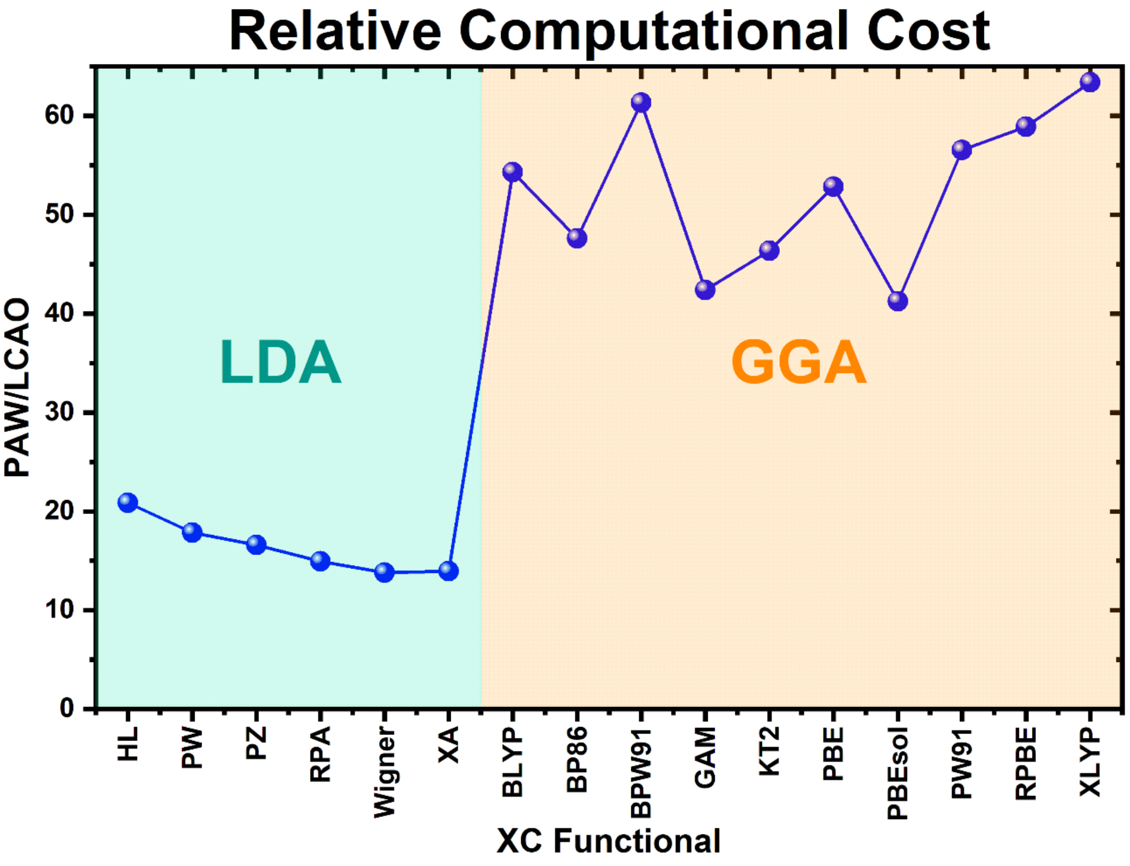

Figure S12 – Relative computational cost between the PAW and LCAO methods, $\frac{\text{PAW}}{\text{LCAO}}$.

To further illustrate this contrast, Figure S12 presents the relative computational cost difference between the two methods, defined as the ratio of the SCF computation time for PAW to

that for LCAO. For LDA functionals, PAW calculations are approximately 14 to 21 times more computationally demanding than their LCAO counterparts. This disparity is even more pronounced for GGA functionals, where PAW requires 41 to 63 times more computational time. These results highlight the significant computational advantage of employing the LCAO method for DFT studies of CBS, particularly in large-scale simulations where efficiency is a critical factor.
